# The zebra finch neuropeptidome: prediction, detection and expression

**DOI:** 10.1186/1741-7007-8-28

**Published:** 2010-04-01

**Authors:** Fang Xie, Sarah E London, Bruce R Southey, Suresh P Annangudi, Andinet Amare, Sandra L Rodriguez-Zas, David F Clayton, Jonathan V Sweedler

**Affiliations:** 1 Department of Chemistry, University of Illinois at Urbana-Champaign, Urbana, IL, 61801 USA; 2 Institute for Genomic Biology, University of Illinois at Urbana-Champaign, Urbana, IL, 61801 USA; 3 Department of Animal Sciences, University of Illinois at Urbana-Champaign, Urbana, IL, 61801 USA; 4 Department of Cell and Developmental Biology, University of Illinois at Urbana-Champaign, Urbana, IL, 61801 USA; 5 Neuroscience Program, University of Illinois at Urbana-Champaign, Urbana, IL, 61801 USA; 6 Beckman Institute, University of Illinois at Urbana-Champaign, Urbana, IL, 61801 USA

## Abstract

**Background:**

Among songbirds, the zebra finch (*Taeniopygia guttata*) is an excellent model system for investigating the neural mechanisms underlying complex behaviours such as vocal communication, learning and social interactions. Neuropeptides and peptide hormones are cell-to-cell signalling molecules known to mediate similar behaviours in other animals. However, in the zebra finch, this information is limited. With the newly-released zebra finch genome as a foundation, we combined bioinformatics, mass-spectrometry (MS)-enabled peptidomics and molecular techniques to identify the complete suite of neuropeptide prohormones and final peptide products and their distributions.

**Results:**

Complementary bioinformatic resources were integrated to survey the zebra finch genome, identifying 70 putative prohormones. Ninety peptides derived from 24 predicted prohormones were characterized using several MS platforms; tandem MS confirmed a majority of the sequences. Most of the peptides described here were not known in the zebra finch or other avian species, although homologous prohormones exist in the chicken genome. Among the zebra finch peptides discovered were several unique vasoactive intestinal and adenylate cyclase activating polypeptide 1 peptides created by cleavage at sites previously unreported in mammalian prohormones. MS-based profiling of brain areas required for singing detected 13 peptides within one brain nucleus, HVC; *in situ *hybridization detected 13 of the 15 prohormone genes examined within at least one major song control nucleus. Expression mapping also identified prohormone messenger RNAs in areas associated with spatial learning and social behaviours. Based on the whole-genome analysis, 40 prohormone probes were found on a commonly used zebra finch brain microarray. Analysis of these newly annotated transcripts revealed that six prohormone probes showed altered expression after birds heard song playbacks in a paradigm of song recognition learning; we partially verify this result experimentally.

**Conclusions:**

The zebra finch peptidome and prohormone complement is now characterized. Based on previous microarray results on zebra finch vocal learning and synaptic plasticity, a number of these prohormones show significant changes during learning. Interestingly, most mammalian prohormones have counterparts in the zebra finch, demonstrating that this songbird uses similar biochemical pathways for neurotransmission and hormonal regulation. These findings enhance investigation into neuropeptide-mediated mechanisms of brain function, learning and behaviour in this model.

## Background

Songbirds, including zebra finches (*Taeniopygia guttata*), are well-established model organisms for a variety of biological functions and are notable for their complex natural behaviours such as vocal communication, learning and social living structures [1-3]. Of particular interest in songbird neurobiology is the set of telencephalic nuclei, referred to collectively as the song control system. This brain circuit is required for vocal learning and song production in male zebra finches and in other songbirds and is also connected to the auditory forebrain lobule that provides the system with auditory information [4,5].

Neuropeptides, a complex group of cell-to-cell signalling molecules, can act as neurotransmitters, neuromodulators, or peptide hormones [6,7]. A few neuropeptides have been previously examined in songbirds [8-15]; those studies demonstrated that neuropeptides could act within brain regions relevant to song and other behaviours. Given the potential for these signalling molecules to impact a wide range of behaviorally-relevant neural functions, the present study aimed to identify a large number of neuropeptides.

Neuropeptide research is complicated by several different factors. Typically, the biosynthesis of neuropeptides starts with the production of a large protein prohormone, which undergoes a variety of processing events before the final products-bioactive peptides-are generated. The gene that codes for a neuropeptide may also contain sequences encoding several other peptides. Peptides can be predicted from prohormone sequences based on common proteolytic cleavage sites [16-19] and directly measured in their bioactive forms from brain samples [20]. The processing of a single prohormone can vary depending on the tissues and/or the developmental stages and, therefore, neuropeptide localization is not always consistent with transcript localization. Consequently, the comprehensive identification, measurement and localization of neuropeptides in any species require a multi-faceted approach.

Taking advantage of the newly-released zebra finch genome sequence [21], we predict, measure and localize the expression of a large complement of neuropeptides in the zebra finch brain using a variety of techniques. A survey of the zebra finch prohormone complement was undertaken using bioinformatic tools. These results were then used to annotate prohormone probes on a widely used zebra finch microarray platform [22]. Neuropeptidomic analyses using previously described mass spectrometry (MS) approaches [20,23-26] were independently conducted to identify the signalling peptides created from these genes in the zebra finch brain and pituitary. *In situ *hybridization (ISH) was performed for a subset of prohormone genes. Both ISH and MS profiling were employed to localize the potential for neuropeptide function in individual song control nuclei. The integration of these different methodologies results in a more comprehensive suite of neuropeptide data that will accelerate investigation into their function in songbirds.

## Results and discussion

### Genomic annotation of neuropeptide prohormone genes

There were 70 matches to known chicken and mammalian neuropeptide prohormone genes in the zebra finch genome resources, resulting in the identification of 51 prohormones with complete sequences. Table 1 provides the predicted zebra finch prohormones and homologous chicken prohormones. Limited homology and genome coverage or assembly errors prevented recovery of the full sequences for some matches. The GenBank zebra finch expressed sequence tag (EST) database was used to confirm the identification and recover sequences. For instance, somastatin (SST) was identified using the EST [GenBank:CK234915] because of insufficient genome coverage and sequencing errors. In other cases, the lack of genome and EST sequences prevented complete recovery of the prohormone. As an example, only a 22 amino acid prediction was obtained for appetite-regulating hormone (ghrelin/obestatin prepropeptide, GHRL) compared to the 116 amino acid chicken GHRL protein sequence.

**Table 1:** Predicted prohormone and other cell-cell signaling peptides.

Prohormone/gene name	Gene symbol	Entrez gene ID	Ensembl_ID	Chicken Entrez ID	Chicken UniProt ID	MS
Adenylate cyclase activating polypeptide 1 (pituitary)	ADCYAP1	100225028	ENSTGUP00000010475	408251	P41534	√
Adrenomedullin	ADM	100231562	ENSTGUP00000008261	423042	na	-
Arginine vasopressin	AVP	100217635	ENSTGUP00000011316	396101	P24787	√
C-fos induced growth factor (vascular endothelial growth factor D)	FIGF	100227068	ENSTGUP00000008337	395255	Q8QGD7	-
C-RF amide peptide	CRF	100226474	na	420716	B0LF68	-
C-type natriuretic peptide 1	CNP1	100226912	na	na	A9CDT5	-
C-type natriuretic peptide 3	CNP3	na	ENSTGUP00000016882	419487	A9CDT6	-
Calcitonin-related polypeptide alpha	CALCA	100228652	ENSTGUP00000008761	396256	P10286	-
CART prepropeptide	CARTPT	100229332	na	na	na	-
Cholecystokinin	CCK	100190220	ENSTGUP00000004812	414884	Q9PU41	√
Chromogranin A (parathyroid secretory protein 1)	CHGA	100225869	ENSTGUP00000012975	423420	na	√
Chromogranin B (secretogranin 1)	CHGB	100230980	ENSTGUP00000002261	421312	na	√
Chromosome 12 open reading frame 39 (spexin)	C12orf39	na	ENSTGUP00000012483	na	na	-
Chromosome 2 open reading frame 40	C2orf40	100225694	ENSTGUP00000010030	771055	na	-
Corticotropin releasing hormone	CRHR1	100190638	ENSTGUP00000011624	404297	Q703P0	√
Endothelin 1	EDN1	100219314	ENSTGUP00000005994	420854	na	-
Endothelin 2	EDN2	na	ENSTGUP00000000838	419559	na	-
Endothelin 3	EDN3	100218153	ENSTGUP00000008244	768509	Q3MU75	-
Galanin prepropeptide	GAL	100229595	ENSTGUP00000010282	423117	P30802	-
Gastric inhibitory polypeptide	GIP	na	ENSTGUP00000014511	419989	A1DPK0	-
Gastrin-releasing peptide	GRP	na	ENSTGUP00000017765	425213	P01295	√
Ghrelin/obestatin prepropeptide	GHRL	na	na	408185	Q8AV73	-
Glucagon	GCG	100226588	ENSTGUP00000014141	396196	P68259	-
Glycoprotein hormones, alpha polypeptide	CGA	100227314	ENSTGUP00000012836	421829	Q6T5C1	-
Gonadotropin-releasing hormone 1 (luteinizing-releasing hormone)	GNRH1	100216318	ENSTGUP00000004488	770134	P37042	√
Growth hormone 1	GH1	100218808	ENSTGUP00000003400	378781	P08998	√
Growth hormone 1 (duplicate on Chr1)	GH1A	100232594	ENSTGUP00000013961	na	na	-
Growth hormone releasing hormone	GHRH	100218168	ENSTGUP00000005071	419178	Q1KNA7	-
Hypocretin (orexin) neuropeptide precursor	HCRT	100225610	ENSTGUP00000018297	374005	Q8AV17	*
Insulin	INS	100231606	ENSTGUP00000009610	396145	P67970	-
Insulin-like 5	INSL5	na	ENSTGUP00000010477	na	na	-
Insulin-like growth factor 1 (somatomedin C)	IGF1	100226283	ENSTGUP00000011633	418090	P18254	-
Insulin-like growth factor 2 (somatomedin A)	IGF2	100144432	ENSTGUP00000009618	395097	P33717	-
Islet amyloid polypeptide	IAPP	100230121	ENSTGUP00000012072	396362	Q90743	-
Mesotocin-neurophysin I	MST	100220528	ENSTGUP00000011315	768516	Q2ACD0	√
Motilin	MLN	100217914	ENSTGUP00000001847	768422	Q9PRP6	-
Natriuretic peptide precursor A	NPPA	100217781	ENSTGUP00000016155	395765	P18908	-
Neuromedin B	NMB	100222573	ENSTGUP00000004989	415333	A0MAR5	-
Neuromedin U	NMU	na	ENSTGUP00000007709	422748	P34963	-
Neuropeptide S	NPS	na	ENSTGUP00000011907	na	na	-
Neuropeptide VF precursor (gonadotropin-inhibitory hormone)	NPVF	100038821	ENSTGUP00000002951	378785	Q75XU6	√
Neuropeptide W	NPW	na	na	769277	na	-
Neuropeptide Y	NPY	100232180	ENSTGUP00000002889	396464	P28673	√
Neurotensin	NTS	100190409	ENSTGUP00000008164	417883	P13724	√
Osteocrin	OSTN	100230749	ENSTGUP00000009566	424907	A5JNH0	-
Pancreatic polypeptide	PPY	na	na	395564	P68248	-
Parathyroid hormone	PTH	100221585	ENSTGUP00000016235	396436	P15743	-
Parathyroid hormone-like hormone	PTHLH	100230763	ENSTGUP00000013060	396281	P17251	√
Platelet derived growth factor D	PDGFD	100221512	ENSTGUP00000013067	418978	na	-
Platelet-derived growth factor alpha polypeptide	PDGFA	100222155	ENSTGUP00000008778	374196	Q90WK2	-
Platelet-derived growth factor beta polypeptide (simian sarcoma viral (v-sis) oncogene homolog)	PDGFB	100228287	ENSTGUP00000010592	374128	Q90W23	-
Prepronociceptin	PNOC	na	na	422019	na	-
Pro-melanin-concentrating hormone	PMCH	100223423	ENSTGUP00000011636	418091	na	-
Prodynorphin	PDYN	na	na	na	na	-
Proenkephalin	PENK	100190068	ENSTGUP00000011453	421131	na	√
Prokineticin 2	PROK2	na	ENSTGUP00000010242	771674	na	-
Prolactin	PRL	na	ENSTGUP00000006544	396453	P14676	√
Prolactin B	PRLB	na	ENSTGUP00000006179	417800	C6ZDB7	-
Prolactin releasing hormone	PRLH	na	na	424018	A3RJ26	-
Proopiomelanocortin	POMC	100219426	ENSTGUP00000017296	422011	Q9YI93	√
Pyroglutamylated RFamide peptide	QRFP	100222487	na	771867	na	-
Relaxin 3	RLN3	100225505	ENSTGUP00000005181	427223	B1AC67	√
Secretin	SCT	100219077	ENSTGUP00000007375	423015	P01280	-
Secretogranin II (chromogranin C)	SCG2	100219534	ENSTGUP00000008135	424808	na	√
Secretogranin V (7B2 protein)	SCG5	100228713	ENSTGUP00000012091	769742	Q5ZI41	√
Somatostatin	SST	100219295	na	396279	P33094	√
Somatostatin 2	SST2	100221267	ENSTGUP00000017225	395106	Q7T2S6	-
Tachykinin, precursor 1	TAC1	100227778	ENSTGUP00000001703	420573	na	√
Thyroid-stimulating hormone beta	TSHB	100220136	ENSTGUP00000000984	395937	O57340	-
Thyrotropin-releasing hormone	TRH	100228179	ENSTGUP00000010894	414344	Q6ZXC3	-
Urocortin	UCN	na	na	na	na	
Urocortin 3	UCN3	100221165	na	na	na	√
Urotensin 2	UTS2	100222633	ENSTGUP00000002613	404535	Q6Q2J6	-
Urotensin 2 domain-containing	UTS2D	100218300	ENSTGUP00000009562	404534	Q6Q273	-
Vascular endothelial growth factor C	VEGFC	100221203	ENSTGUP00000006624	422573	O57352 (quail)	-
Vasoactive intestinal peptide	VIP	100217965	ENSTGUP00000011533	396323	P48143	√

The list of 76 prohormone and signalling genes predicted from the survey of the zebra finch genome; information on gene name and identification are provided. In the mass spectrometry (MS) column, a check mark (√) indicates confirmation via MS, an asterisk (*) indicates their homologues are identified in other species and a dash (-) indicates no confirmation. Peptides from 24 predicted genes were detected by MS. The protein sequences of the discovered prohormone and signaling genes are provided in FASTA format [see Additional File 2].

Slight differences in assembly releases resulted in the incomplete presence of nociceptin (PNOC) and pancreatic polypeptide (PPY) prohormones. An EST [Genbank:CK234392] was matched to the chicken PNOC and translation of the EST recovered the first 77 amino acids. This EST was not present in the genomic data as only 35 bases were matched in the trace archives.

A match for PPY was identified in the pre-release assembly but not in the release assembly. However, there was no supporting EST data. The complete chicken PPY prohormone was reported in UniProt but was not present in the available chicken genome. Peptide sequences have also been reported in UniProt for the gull, turkey and ostrich, implying that the zebra finch version may be present.

Using the EST database and chicken data, possible alternative splicing was detected for six genes.

Three prohormones - pituitary adenylate cyclase-activating polypeptide (ADCYAP1), glucagon (GLUC) and vasoactive intestinal peptide (VIP) - have been reported with alternative isoforms in chicken. Tachykinin 1 (TAC1) has multiple mammalian isoforms and two zebra finch isoforms were identified and subsequently confirmed by ESTs. However, although no chicken TAC1 isoforms have been reported, four TAC1 chicken isoforms are predicted in the corresponding National Center for Biotechnology Information gene entry. Two prohormones, augurin or chromosome 2 open reading frame 40 (C2orf40) and urotensin 2 domain containing (UTS2D), had a single isoform that was supported by EST data. In both cases, an alternative sequence was predicted using the chicken sequence with Wise2 [27].

The zebra finch prohormone complement is similar to the chicken and to mammals, with evidence for 68 prohormone homologues in either or both the avian and mammalian genomes. This included six prohormones that matched the chicken genome reported by Delfino *et al*. [28]. Urocortin 1 (UCN), identified in the zebra finch by EST [GenBank:DV950835], was not identified in the chicken genome. However, UCN still may be present because there were gaps in the chicken genome between the flanking genes. A proenkephalin-B prohormone (prodynorphin, PDYN) similar to mammals was found in the zebra finch genome but no match was found in the chicken genome or related resources [28].

There was no evidence for three chicken prohormones in the zebra finch genome: apelin (APEL), renal natriuretic peptide (RNP) and gonadoliberin II (GNRH2). APEL has been reported in mammals and identified in chicken by Delfino *et al*. [28]. There were no suitable matches to chicken RNP, a member of the natriuretic family, in either the zebra finch or mammals, indicating that this duplication may have occurred after songbirds (order: Passeriformes) diverged from chickens (order: Galliformes). There was no match to mammalian GNRH2 and the chicken GNRH2 was only reported as a protein sequence with no corresponding location on the chicken genome.

Two prohormones, C-type natriuretic peptide 1 (CNP1) and corticotrophin-releasing factor (C-RF) amide peptide (CRFamide), were only found in avian genomes. CNP1 appears to be an avian-specific duplication, occurring after the divergence from mammals. An RF-amide similar to prolactin-releasing peptide prohormone, CRFamide was also identified in chicken and mammalian genomes; it had a high conservation of the 20 amino acid prolactin-releasing peptide found in the mammalian prolactin-releasing hormone prohormone.

Twenty-three known prohormones were not found in the chicken or zebra finch genomes; 18 of the 23 appear to belong to gene families where at least one member is present in both mammalian and avian genomes. These may result from a duplication in mammals that occurred after the avian and mammalian species diverged. For at least the natriuretic family, there are both avian-specific and mammalian-specific duplications. One of these, the gene-regulated endocrine-specific protein 18 (RES18), is known to be in Eutherian mammals. The lack of matches to the remaining prohormones may be explained by limited homology with confounding factors caused by incomplete genome sequencing coverage which thereby prevented a reliable prediction. For example, no evidence for proprotein convertase subtilisin/kexin type 1 inhibitor (PCSK1N) was identified in the avian genome although Kudo *et al*. [29] reported low homology between mammalian sequences and *Xenopus *and zebrafish (*Danio rerio*) sequences.

### Identification of other signaling genes

Other signalling prohormones, including prolactin (PRL) prolactin B (PRLB) and insulin growth factor-2 (IGF2), were also identified. In addition, secretogranin V or 7B2 protein (SCG5) was identified, which is essential for prohormone convertase 2 (PCSK2) function [30-32]. Our genomic survey also confirmed duplication of somatotropin or growth hormone (GH) on chromosomes 1 and 27, which was also supported by EST data [33].

### MS-based detection and identification of neuropeptides in brain and pituitary

MS can directly measure peptides without prior knowledge of the sequences of the prohormone or the expected peptides. We implemented two complementary MS platforms because this combined approach has been shown to provide a more complete list of peptides [24,34,35]. A total of 90 peptides were characterized from the zebra finch brain and pituitary and 95% of these peptide sequences were confirmed by tandem MS (MS/MS) (See Additional File 1 for the sequences and masses of identified peptides). We assigned the MS/MS spectral information from the peptides characterized via MS to our database of prohormones. This allowed us to annotate our MS-confirmed peptide sequence information as peptide products of 24 unique prohormones and other signaling proteins (See Table 1). Every individually detected and sequenced peptide was counted in the present study.

The peptides we detected represent peptides processed from the prohormones; most were produced by cleavage at basic sites. However, because some peptides require processing at unconventional cleavage sites, they may not be predicted from the primary structures of the prohormones using bioinformatics tools such as NeuroPred [17]. For example, five chromogranin A (CHGA) peptides-WNKMDEL, WNKMDELA, WNKMDELAKQL, WNKMDELAKQLT and WNKMDELAKQLTS-were all sequenced by MS/MS independently and, thus, were considered as five peptides in our total count. Similar examples of truncated peptides were detected for neurotensin (NTS), cholecystokinin (CCK), proenkephalin A (PENK), secretogranin-1/chromogranin B (SCG1), secretogranin II/chromogranin C (SCG2), SCG5, thymosin-beta and cerebellin (CBLN1), either from the *C*-terminus or from the *N*-terminus. Each of these was counted as a distinct peptide because similar truncated peptides in other species have been reported to have biological activity. For example, several different CBLN1 peptides have been described in other animals. Two of these, the cerebellin hexadecamer and a truncated des-Ser^1 ^pentadecamer peptide, are both endogenous peptides with biological relevance in rodent studies [36]. In addition, two more cerebellin-related peptides have been recently described that lack one residue at the *C*-terminus of cerebellin and des-Ser^1^-cerebellin, respectively [37].

Although several of the detected peptides may represent extracellular degradation that can occur during acid extraction or postmortem decay, rather than naturally processed bioactive peptides, our rapid dissection technique and use of chilled acetone minimizes the potential for post-dissection proteolysis. Furthermore, the truncated peptides usually eluted from the liquid chromatography column at different retention times, indicating they were formed prior to the MS procedure. Given our prior experience with peptide isolation, we surmise that most of the peptides detected were derived from endogenous proteolytic processing.

Sequenced peptides directly detected in the brain assisted in the identification and confirmation of the correct sequence in the zebra finch genome. Many neuropeptides are well conserved across species, especially between avian species. For example, the NTS peptide in chicken is QLHVNKARRPYIL; the predicted zebra finch peptide sequence based on the genomic assembly is QLHVNKSRRPYIL, which has an A to S substitution at the seventh amino acid residue. However, our MS analysis found that the peptide sequence in zebra finch was the same as that in chicken. Comparison to published zebra finch ESTs, and other genomic databases, indicates that this is most likely an assembly error rather than a single nucleotide polymorphism in the zebra finch genome. The trace archive files also support the MS sequence.

When searching the MS data against the zebra finch resources and the prohormone databases of other species, additional peptides were identified in the zebra finch. These include: SKAGRVKLPP from mitochondrial ribosomal protein S26 (MRP S26), LPECCRQKTCSCRIYDLLHGMGNHAAGILTL-amide from orexin (OREX), SGSAKVAFSAIRSTNH and SGSAKVAFSAIRSTN from CBLN1 and PVDLAKWDGPSLS from phosphatidylethanolamine binding protein 1 (PEBP1).

Peptides from non-prohormone proteins were also detected with MS. Several thymosin-beta peptides, including Ac-SDKPDMAEIEKFDKSKLKKTETQEKNPLPSKETIEQEKQAGES, Ac-SDKPDMAEIEKFDK, Ac-SDKPDMAEIEKFD and Ac-SDKPDMAEIEKF, were identified in the zebra finch brain. Thymosin-beta is commonly observed in the brain [34,35,38] and is observed with neuropeptides during stimulated neuropeptide release [39,40]. Three peptides, TVGDVNTERPGMLDF, KQATVGDVNTERPGMLDF and Ac-SEAAFQKAAEEVKQL from carboxypeptidase N, polypeptide 2 (CPN2), were also identified in the zebra finch. CPN2 is the regulatory subunit of a secreted tetrameric protein expressed in the nervous system of other animals [41]; its identification here illustrates the power of MS to detect other unusual protein processing products in the brain.

### Discovery of novel peptides

**Figure 1:**
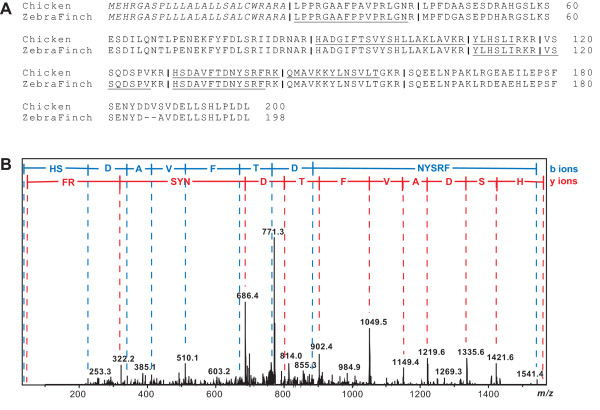
**The vasoactive intestinal peptide (VIP) prohormone has been characterized in the finch**. (A) Aligned zebrafinch and chicken VIP prohormones show peptides (underlined) and prohormone cleaveages ("|" symbol) and highlight the few differences between these two species. (B) The VIP peptide HSDAVFTDNYSRF has been confirmed via tandem mass spectrometry. The peptide HSDAVFTDNYSRF was fragmented in the mass spectrometer. Two different fragment ion series (b- and y-ions) were obtained, depending on whether the charge was carried on the *N*-terminal or *C*-terminal side of the cleavage site. The amino acid residue(s) were assigned based on the mass difference between two peaks, as annotated by the blue and red letters. Based on this information, the sequence of this VIP-related peptide is unambiguously determined.

Using MS approaches, we directly detected several novel VIP and ADCYAP1 peptides in addition to the previously described peptides. Specifically, the MS data showed strong evidence for the VIP peptide HSDAVFTDNYSRF (Figure 1) and the ADCYAP1 peptides, VGGASGGLGDDAEPLT, HIDGIFTDSYSRY and QMAVKKYLAAVLamide in the zebra finch brain. These novel peptides overlap with the well-characterized longer VIP and ADCYAP1 peptides, but are processed at basic sites that appear not to have been reported previously for VIP and ADCYAP1 in most other species. The zebra finch peptides were shorter than the VIP, PACAP-27 and PACAP-38 peptides described in rat and mouse [42,43], suggesting that VIP and PACAP prohormones may be subject to different processing pathways in the zebra finch. The VIP, PACAP-27, PACAP-38 peptides are neurotransmitters of the inhibitory nonadrenergic, noncholinergic nervous system involved in a number of physiological conditions, mediated through common VIP/ADCYAP1 (VPAC_1 _and VPAC_2_) receptors and specific ADCYAP1 (PAC_1_) receptors [42,43]. The newly-discovered short VIP and ADCYAP1 peptides may also interact with these receptors or have their own mechanisms of action to be revealed in future experiments.

### Characterization of posttranslationally-modified peptides

Posttranslational modifications (PTMs) can alter the biological activity of peptides. They can be detected using MS because each PTM has a characteristic mass shift. These PTMs can provide more resistance to enzymatic degradation and regulate the binding affinity to receptors and, thus, directly impact the bioactivity of peptides. Some common PTMs, including *C*-terminal amidation, disulfide bonds, *N*-terminal pyroglutamate formation, and *N*-terminal acetylation, were identified in the zebra finch peptides (see Additional File 1). For example, the *C*-terminal amidation of the LPXRF-amide (X = L or Q) motif of NPVF peptides and the disulfide bond of the CYIQNCPXG-amide (X = any amino acid) motif of Arg-vasopressin (AVP) were detected in this study. These evolutionarily conserved PTMs may be essential for the interaction of peptides with their cognate receptors across Metazoan.

### Distribution of prohormone gene expression in adult brains

In addition to understanding the peptide complements, the locations of expression also impact biological function. To identify brain regions that express prohormone-related genes, ISH was conducted for 15 genes in adult zebra finch brains-12 prohormone genes and CBLN1, phosphatidylethanolamine binding protein 1 (PEBP1) and, lastly, CPN2, which was used as a control because it was detected in the MS experiments (Table 2 and Table 3). The ESTs used as ISH riboprobe templates covered at least 50% of the mRNAs as predicted by the Ensembl gene models (release 55; http://www.ensembl.org/Taeniopygia_guttata). Each EST showed a homology of at least 79% to the corresponding chicken mRNA sequence. Sense negative control hybridizations showed no specific label, demonstrating the high stringency of the hybridization conditions and suggesting high specificity of the riboprobes to the zebra finch transcripts. With the exception of the sexually dimorphic song nuclei, no sex differences in distribution were detected.

**Table 2:** Gene expression distributions characterized via *in situ *hybridization.

Gene name	Gene symbol	Entrez gene ID	Brain region										MS
				
			Area X	LMAN	HVC	RA	ME/PIT	POA	HP	S	TN	MHYP	
Adenylate cyclase activating polypeptide 1 (pituitary)	ADCYAP1	100225028	-	√	-	-	√	√	√	√	√	√	√
Neuropeptide VF precursor (gonadotropin-inhibitory hormone)	NPVF	100038821	-	-	-	-	-	-	-	-	-	√	√
Proenkephalin	PENK	100190068	-	√	-	-	√	√	-	-	-	√	√
Carboxypeptidase N, polypeptide 2	CPN2	100222124	√	√	√	√	-	-	√	-	-	-	*
Chromogranin B (secretogranin 1)	CHGB	100230980	√	√	√	√	-	√	√	√	-	√	√
Hypocretin (orexin) neuropeptide precursor	HCRT	100225610	√	-	√	-	-	-	-	-	-	√	*
Mesotocin-neurophysin I	MST	100220528	√	√	√	√	-	√	√	√	√	√	√
Neuropeptide Y	NPY	100232180	√	√	√	-	√	√	-	√	-	√	√
Neurotensin	NTS	100190409	√	√	√	-	-	√	-	-	-	√	√
Proopiomelanocortin	POMC	100219426	√	√	√	√	√	-	-	-	-	-	√
Somatostatin	SST	100219295	√	√	-	√	√	√	-	-	√	√	√
Tachykinin, precursor 1	TAC1	100227778	√	-	√	-	-	√	-	√	-	√	√
Vasoactive intestinal peptide	VIP	100217965	√	√	√	-	√	-	-	√	√	√	√
Cerebellin 1 precursor	CBLN1	unpredicted	√	-	√	√	-	√	√	√	-	√	*
Phosphatidylethanolamine binding protein 1	PEBP1	100190590	√	√	-	√	-	√	√	-	√	√	*

The distributions of 15 genes have been determined via *in situ *hybridization (ISH), with the distribution of expression being highlighted in four major song nuclei (Area X, LMAN, HVC, RA) and other behaviorally-relevant brain areas. For each specific brain region, a check mark (√) indicates detected by ISH and a dash (-) indicates no expression as characterized by ISH. In the mass spectrometry (MS) column, a check mark (√) indicates confirmation via MS and an asterisk (*) indicates their homologues are identified in other species. Abbreviations used for non-song control brain areas: ME/PIT-median eminence or pituitary; POA-preoptic area; HP-principle cells of the hippocampus; S-septal nuclei; TN-nucleus taeniae; MHYP-ventromedial hypothalamic nucleus or paraventricular nucleus.

**Table 3:** Microarray and *in situ *hybridization (ISH) results for prohormone expressed sequence tags (ESTs).

Prohormone/gene name	Gene symbol	**Accession No**.	AASS *P*-value	SASS *P*-value	AASA *P*-value	ISH *P*-value
Adenylate cyclase activating polypeptide 1 (pituitary)	ADCYAP1	CK303710	0.46	0.64	0.78	-
Adenylate cyclase activating polypeptide 1 (pituitary)	ADCYAP1	CK303710	0.91	0.56	0.64	-
Adrenomedullin	ADM	DV955971	**<0.01**	0.67	**<0.01**	0.58
C-RF amide peptide	CRF	DV949637	0.69	0.43	0.69	-
Cholecystokinin	CCK	CK302967	**<0.01**	0.72	**<0.01**	**0.05**
Chromogranin A (parathyroid secretory protein 1)	CHGA	DV950480	0.07	0.06	0.96	-
Chromogranin B (secretogranin 1)	CHGB	CK308123	0.14	0.22	0.79	-
Chromogranin B (secretogranin 1)	CHGB	CK308816	0.01	0.14	0.21	-
Chromosome 2 open reading frame 40	C2orf40	CK314083	0.10	0.19	0.72	-
Corticotropin-releasing hormone	CRHR1	CK309161	0.05	0.02	0.61	-
Endothelin 3	EDN3	DV946954	0.84	0.47	0.61	-
Galanin prepropeptide	GAL	DV957373	0.23	0.24	0.03	-
Gastrin-releasing peptide	GRP	CK312472	0.02	0.23	0.20	-
Glycoprotein hormones, alpha polypeptide	CGA	CK310677	0.77	0.94	0.83	-
Growth hormone 1 (Chr27)	GH1	CK301315	0.04	0.20	**<0.01**	**0.04**
Growth hormone 1 (duplicate on Chr1)	GH1A	DV950694	0.42	0.11	0.40	-
Hypocretin (orexin) neuropeptide precursor	HCRT	DV955493	0.06	0.42	0.26	-
Insulin-like growth factor 1 (somatomedin C)	IGF1	DV948990	**<0.01**	0.14	0.01	0.11
Islet amyloid polypeptide	IAPP	CK234383	0.57	0.57	0.27	-
Neuromedin B	NMB	DV950473	0.82	0.41	0.30	-
Neuromedin B	NMB	DV959623	0.71	0.03	0.07	-
Neuropeptide W	NPW	DV945212	0.61	0.19	0.41	-
Neuropeptide W	NPW	DV945212	0.34	0.22	0.78	-
Neuropeptide Y	NPY	CK310313	**<0.01**	0.28	**<0.01**	0.54
Neurotensin	NTS	CK302282	0.06	0.11	**<0.01**	**0.06**
Neurotensin	NTS	CK305091	0.01	0.11	**<0.01**	-
Platelet-derived growth factor alpha polypeptide	PDGFA	DV959989	0.06	0.88	0.04	-
Prepronociceptin	PNOC	CK234392	0.06	0.03	**<0.01**	-
Proenkephalin	PENK	CK301334	0.08	0.68	0.04	-
Proenkephalin	PENK	CK312129	0.16	0.94	0.18	-
Secretogranin II (chromogranin C)	SCG2	CK313614	0.86	0.27	0.35	-
Secretogranin II (chromogranin C)	SCG2	DV945506	0.50	0.14	0.39	-
Secretogranin V (7B2 protein)	SCG5	CK313144	0.21	0.04	0.36	-
Somatostatin	SST	CK234915	**<0.01**	0.65	**<0.01**	-
Somatostatin 2	SST2	CK314968	0.71	0.11	0.05	-
Tachykinin, precursor 1	TAC1	CK301285	0.01	0.73	0.03	-
Tachykinin, precursor 1	TAC1	DV958953	0.18	0.20	0.02	-
Thyrotropin-releasing hormone	TRH	CK302320	0.18	0.20	0.95	-
Urocortin	UCN	DV950835	0.336	0.285	0.908	-
Urotensin 2 domain containing	UTS2D	DV945629	0.11	0.10	0.99	-
Vasoactive intestinal peptide	VIP	DV958568	0.07	0.07	0.94	-

Forty-one unique ESTs that represent 32 prohormone genes were identified on the zebra finch brain 20K SoNG microarray. Some ESTs were spotted twice on the array, and in some cases more than one EST are from a single prohormone; all ESTs are included. Listed are the raw *P*-values from an experiment that compared expression profiles in the adult male auditory forebrain after birds were exposed to three auditory stimuli: (AA, indicating the bird was habituated to song A on one day then tested for its response to the song on the next day), novel song (SA, indicating the bird heard silence on the first day and then song A for the first time on the day of testing) or silence (SS, indicating the bird heard silence both days) [44]; a false discovery rate (FDR) significant cutoff of FDR < 0.05 was used to select genes for ISH analysis. Comparisons are: birds that heard either familiar song versus those that heard silence (AASS), birds that heard novel song versus those that heard silence (SASS), or birds that heard familiar song versus those that heard novel song (AASA). Six ESTs that were reported to show a significant change in the familiar group [44] were used as riboprobes for ISH analysis. Of these six, three ESTs showed a significant change in the number of cells with modulated expression levels related to song experience. Significant *P*-values (*P *< 0.01 for microarray; *P *< 0.05 for ISH values) are in bold. A dash (-) indicates that the EST was not investigated using ISH.

**Figure 2:**
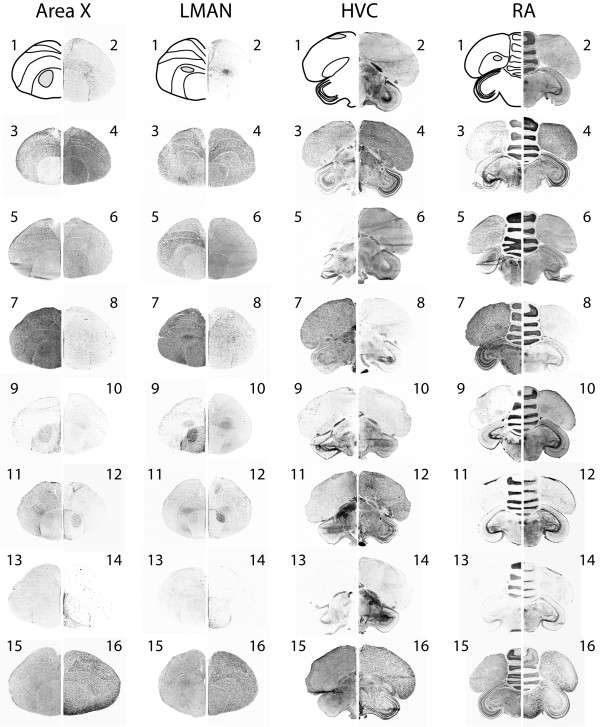
**Distribution of genes in four major song nuclei**. Results of *in situ *hybridization for 15 selected genes in four major telencephalic song nuclei: Area X, the lateral magnocellular nucleus of the anterior nidopallium (LMAN), HVC, and the robust nucleus of the arcopallium (RA), showing a complex distribution of expression across the song system. The approximate area of each nucleus is illustrated in the top left panel displaying results for each nucleus. Left and right hemispheres display different genes, all labelled with numbers as follows: 1-schematic; 2-NTS; 3-ADCYAP1; 4-VIP; 5-SCG1; 6-OREX; 7-MST; 8-POMC; 9-NPY; 10-CPN2; 11-CBLN1; 12-TAC1; 13-NPVF; 14-PENK; 15-PEBP1; 16-SST.

Expression of the 15 genes was examined in the four major telencephalic song nuclei: Area X, the lateral magnocellular nucleus of the anterior nidopallium (LMAN), HVC (used as a proper name) and the robust nucleus of the arcopallium (RA). Three genes, CPN2, MST and SCG1, were expressed in all four nuclei (Table 3, Figure 2), but POMC and NPVF mRNAs were not detectable in the song nuclei. The remaining 10 genes were expressed in a subset of the song control nuclei (Table 3, Figure 2).

Neuropeptides also modulate physiological processes and behaviours other than song. ISH demonstrated that prohormone genes were expressed in brain areas involved in controlling a variety of processes (Table 1). For example, we found hybridization for 13 of the 15 genes within the paraventricular nucleus, ventromedial nucleus and the preoptic area of the hypothalamus, brain areas that are involved in regulating reproduction. Several of these genes were also expressed within the pituitary or the hypothalamic gateway to the pituitary, the median eminence. These structures are essential for reproductive control and other basic physiology such as stress responses and regulation of thyroid function. The septal nuclei and nucleus taeniae, implicated in affiliative and aggressive behaviours, showed some low levels of prohormone labelling. In addition, the principal cells of the hippocampus, a structure required for spatial learning, showed hybridization with several prohormone riboprobes and CPN2. Images of sections throughout the entire adult male brain processed with ISH can be found at http://neuroproteomics.scs.illinois.edu/songbird/neuroanatomy.html.

### Analysis of song-regulated prohormone genes

From the genome-wide survey of zebra finch prohormones, the most commonly used zebra finch microarray platform (20K SoNG microarray) was annotated for its prohormone content [22]. There were 40 probes corresponding to 31 prohormone genes, including three probes corresponding to the duplicated GH genes and SST2 identified on the array (Table 3). We then reexamined data from an experiment where this microarray was used to characterize changes in gene expression in the adult zebra finch auditory forebrain during the phenomenon of song response habituation [44]. In this data set, we identified six prohormones that showed a significant decrease in expression levels after song habituation (false-discovery rate adjusted p-value < 0.05) [45].

We therefore performed ISH using ESTs for these six genes on brains from birds that experienced either silence (no playback of any song), playback of a novel song or playback of a familiar (habituated) song (Table 3). Two of these genes, CCK and GH (chromosome 27 gene), showed significant changes (*P*-value = 0.051 and *P*-value = 0.036, respectively) in the number of cells above the intensity threshold in the auditory forebrain of birds that heard familiar song compared to those that heard novel song or no song. GH showed the decrease in expression expected from the microarray results. However, CCK actually showed an increase. NTS showed a strong trend towards a lower number of labelled cells in the auditory forebrain lobule after hearing familiar song (*P*-value = 0.057). Insulin-like growth factor 1 (IGF1), adrenomedullin (ADM), and neuropeptide Y (NPY) did not show a significant difference in the number of labelled cells in the auditory forebrain lobule across song exposure conditions

### Peptide profiling in the song nuclei

**Figure 3:**
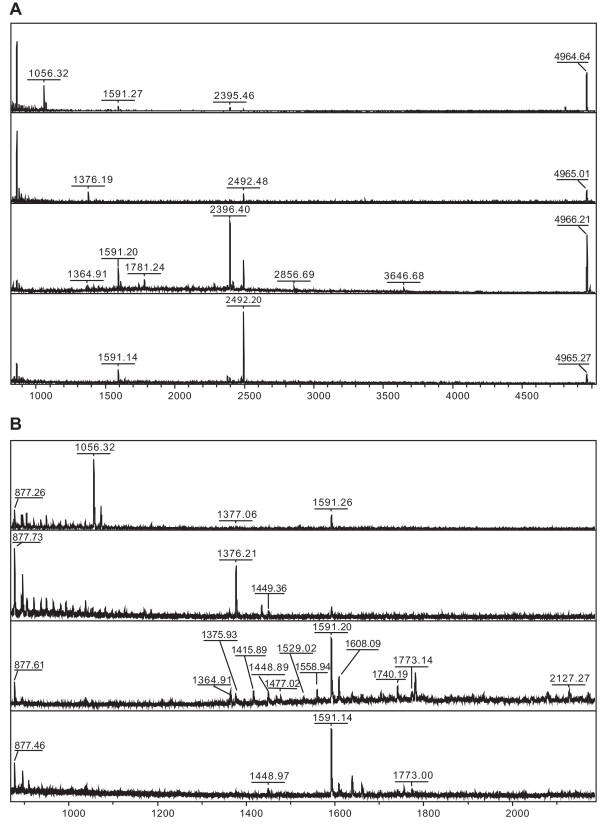
**Peptide profiling of the four major song nuclei by mass spectrometry**. The homogenates of four major song nuclei (from top to bottom: LMAN, Area X, HVC, and RA) were analysed by MALDI-TOF MS. (A) Peptide profile of four song nuclei in the *m/z *range of 870-5000. (B) Magnified spectrum between 870-2200 *m/z*. The individual peaks were mass-matched to peptides previously shown to be present in the brain via tandem mass spectrometry.

In order to directly measure a subset of the peptides that exist within the major telencephalic song nuclei, we conducted MS analysis on brain punches of Area X, LMAN, HVC and RA. The quantity of peptides in these areas was not adequate for MS/MS analysis to determine the amino acid sequences. Thus, we used matrix-assisted laser desorption/ionization time-of-flight (MALDI-TOF) MS to generate the peptide profile of each nuclei homogenate. We then assigned the peaks in the spectra based on mass matches to the peptide list generated in the peptidomic study of the whole brain. Figure 3 shows the spectra from four different song nuclei, demonstrating that each nucleus is characterized by slightly different peptide profiles. Thirteen peptides were putatively identified in HVC by mass match, and a subset of those was also detected in other song nuclei (Table 4).

**Table 4:** Identification of peptides in the four major song nuclei.

Mass	Putative Identity	Prohormone	Detected			
			
			Area X	LMAN	HVC	RA
877.4	YGGFMSSE	POMC	√	√	√	√
					
	YGGFMRF	PENK				

1375.9	RPRPQQFFGLMamide	TAC1	√	√	√	-
					
	WNKMDELAKQL	CHGA				

1415.8	SEYRGHLPAEKE	SCG1	-	-	√	-
					
	VGGASGGLGDDAEPLT	ADCYAP1				

1448.8	VGRPEWWLDYQ	PENK	√	-	√	√

1477.0	WNKMDELAKQLT	CHGA	-	-	√	-

1529.0	GKTNRYDVERQY	SCG1	-	-	√	-

1558.9	HSDAVFTDNYSRF	VIP	-	-	√	-

1591.2	p-QLHVNKARRPYIL	NTS	√	√	√	√

1608.1	MPLGHYEDSSRDSPF	SCG2	√	√	√	√

1740.2	NPGKTNRYDVERQY	CHGB	-	-	√	-

1773.1	SVNPYLQGQRLDNVVA	SCG5	-	-	√	√

2127.5	DSAGEQLPEGREEGRPALSE	CHGB	-	√	√	-

4966.1	Ac-SDKPDMAEIEKFDKSKLKKTETQEKNPLPSKETIEQEKQAGES	TMSB	√	√	√	√

The mass listed is the observed mass [M+H]^+ ^from the MALDI TOF mass spectrometry of the HVC samples. The peptide identities of 13 peaks in the mass spectrum of the HVC homogenate were assigned based on mass matches to the previously identified peptides. Three peaks (877.4, 1375.9, and 1415.8 *m/z*) were assigned to two putative peptides each. A check mark (√) indicates the song nucleus where the masses were observed. A dash (-) indicates it was not detected.

Both MS analysis and ISH of individual song nuclei demonstrated that the peptides may have complex distributions across the song system, sometimes present in all nuclei but usually in a subset. Not all peptides were measured by both techniques; but for those that were, several complexities inherent to neuropeptide characterizations were observed. First, we again saw multiple peptides from the same prohormone but not all of the peptides were detected in the same brain areas. For example, some SCG1 peptides were detected within HVC only, while others were measured within both HVC and RA. This may indicate specific processing of individual peptides that get targeted to each brain area. Second, the distribution of the mRNA did not always predict where the peptides were detected, demonstrating that cell bodies that express prohormone genes may be in different neuroanatomical locations than the cellular projections in which the peptides reside [12,46,47]. Again, for SCG1, ISH showed expression in all four song nuclei but a more restricted peptide distribution was demonstrated using MS. This may reflect a mechanism of controlled peptide transport and release or a mechanism by which prohormone gene transcription is regulated independently from the translation and cleavage of active peptides. It is theoretically possible that peptides and mRNAs were not co-localized due to sensitivity limitations of one of the techniques, but the proposed biological interpretations are consistent with known peptide functions/processes. In addition, the neuroanatomical distribution of prohormone gene expression is consistent with previous gene expression findings in HVC [48,49] and largely with previous immunohistochemical analysis of peptide distribution in song nuclei [12,46]. The distribution of peptides and genes did not neatly separate according to brain region (for example, striatal (Area X) and pallial (HVC, RA, LMAN)) or function (for example, LMAN and X are part of a functionally distinct part of the song circuitry from HVC and RA). The song system is, however, clearly a circuit in which specific neuropeptide signaling could modulate behaviour.

### General summary

Neuropeptides are important signalling molecules that modulate a variety of physiological and behavioural processes. In songbirds such as the zebra finch, neuropeptides have the potential to influence a complex behaviour of particular interest-song. As few neuropeptides had been previously investigated in songbirds, we endeavored to identify as many potential neuropeptides as possible, using the newly released zebra finch genome to annotate our efforts. Because multiple neuropeptides can be produced from a single prohormone and can be transported distally to the location of the cell body, we employed several complementary techniques. Specifically, we combined a bioinformatics approach to whole-genome prohormone gene prediction, direct measurement of neuropeptides within the whole brain and within specific brain areas specialized for song, and neuroanatomical gene expression mapping for a subset of prohormone genes. Employing this multi-faceted approach, we identified 90 peptides, including several novel neuropeptides, directly from the whole brain and described the potential for different peptide profiles to exist in various brain regions such as the song control nuclei.

Furthermore, we identified 70 putative prohormones in the zebra finch. Most prohormone genes have brain EST support. We also identified several new prohormones that were not assigned by official gene models through the Ensembl pipeline or described in other species [21]. Several prohormone genes showed the potential for alternative splicing, indicating that neuropeptide signaling may be quite complex. Although some genes, typically a subset of genes that belong to a multi-gene family, are absent in the zebra finch, the complement of zebra finch prohormone genes is similar to that in the chicken and mammals [50]. Given that only a small number of neuropeptides have been investigated in songbirds to date [8-15,51], this genomic analysis greatly expands the opportunity to investigate how the regulation of prohormone genes contributes to neural function.

Several peptides can be produced from one prohormone gene and, using our whole-genome prohormone gene predictions, we often annotated multiple peptides as belonging to the same gene. We considered each of these peptides to be distinct, even if they were a series of truncated forms from one peptide, because even these seemingly small changes may have biologically relevant consequences. We also detected several novel peptides from the zebra finch brain that showed sequence homology to VIP and ADCYAP1 prohormones, which may have novel mechanisms of action.

Due to the impact neuropeptides have on animal physiology and many complex natural behaviours [8-15,51], we were particularly interested in characterizing the peptide profiles in the four major song control nuclei in the zebra finch: Area X, LMAN, HVC and RA. We also identified prohormone expression in several brain areas important for regulation of essential physiological processes - learning and memory, reproduction and other social behaviours. It is possible that neuropeptides processed from these genes act at cell terminals located in a brain area other than where the cell bodies measured with ISH reside. For example, NPY fibers were described in the songbird hippocampus [10,52], but we did not detect labelling there with ISH. Several of our findings are, however, consistent with previous reports. For example, VIP and mesotocin levels in septal nuclei, where these mRNA were localized, are regulated by social behaviours in songbirds and are generally correlated with levels of sociality in the zebra finch and related species [53-56]. Many of the prohormones investigated here have not been functionally tested in the songbird; thus, neuroanatomical localization of prohormone expression is a useful guide for further examination of the function of neuropeptides in songbird biology.

In order to further delve into the potential for neuropeptides to play a functional role in zebra finch behaviour, we used our genome-wide prohormone gene annotation to identify prohormone ESTs (Table 3) on the zebra finch brain SoNG microarray [22]. Previously published experiments that compared gene expression patterns across brain regions, sex and age, or experience reported differences in some of these ESTs, including a change in proenkephalin in HVC after birds sang [22,44,48,57,58]. Here, we focused on genes that showed changes during song response habituation in the auditory forebrain lobule, an area crucial for processing and learning complex, salient sounds [44,59-62]. In our annotations, at least six prohormone genes showed a significant decrease in expression levels 24 h after a zebra finch was entrained by song repetition and our ISH experiments confirm half of these changes [44]. These results provide an example of how prohormone gene expression can be affected by behavioural paradigms relevant to song learning [44,61,62].

## Conclusions

The most obvious outcome from this study is that the zebra finch peptidome and prohormone complement is now well characterized; this will become an important resource for a number of follow-up studies. The combination of bioinformatic prediction of prohormone genes, direct measurement of peptides and neuroanatomical localization of prohormone gene expression provides comprehensive and compelling insights into the influence of neuropeptides on songbird brain function and behaviour.

## Methods

### Animals

We used developing (posthatch day 1-45) and adult male and female zebra finches bred and raised in an aviary at the Beckman Institute animal facility, University of Illinois, Urbana-Champaign. All procedures involving animals were preformed in accordance with protocols approved by the University of Illinois, Urbana-Champaign Institutional Animal Care and Use Committee.

### Chemicals

Acetic acid, magnesium chloride (MgCl_2_), and 2,5-dihydroxybenzoic acid (DHB) were purchased from Sigma-Aldrich Chemical Co. (MO, USA). Water was prepared by a Milli-Q filtration system (Millipore, MA, USA). Hydrochloric acid (HCl) and high-performance liquid chromatography (HPLC)-grade solvents were purchased from Fisher (NJ, USA). Trifluoroacetic acid (TFA) was purchased from Pierce Biotechnology, Inc. (IL, USA). Heptafluorobutyric acid (HFBA) was purchased from Thermo (IL, USA). Formic acid (FA) was purchased from Fluka (WI, USA). The mixture of standard peptides used for the external calibration of MALDI-TOF MS was purchased from Bruker Daltonics (MA, USA).

### Neuropeptide prohormone identification and characterization

The bioinformatics identification of zebra finch prohormone genes was conducted with two sets of candidate prohormone gene lists using the approach described by Southey *et al*. [19,50]. The initial list of candidate prohormone genes was derived from known mammalian genes supplemented by known or homologous avian genes identified by Delfino *et al*. [28]. The second candidate list of homologous chicken or mammalian genes that matched peptide sequences were obtained by *de novo *sequencing. Candidate genes were searched for in the zebra finch genome resources including genome (assembly build version 1.1), whole genome trace archives and EST databases.

### Extraction of peptides

Optimized sampling procedures were used for peptide extraction [20,34]. Zebra finch brains were dissected from the skull and immediately homogenized in cold acidified acetone (40:6:1 acetone:H_2_O:HCl, v/v/v) on a bed of ice. Following centrifugation at 14,000 rpm for 30 min at 4°C, the supernatant was removed, dried in a SpeedVac (Savant Instruments, NY, USA) and reconstituted in a solution containing 95% H_2_O/5% CH_3_CN/0.1% TFA. The sample was then filtered by a Microcon YM-10 unit (10 kDa molecular weight cut-off, Millipore, MA, USA).

### Liquid chromatography fractionation

Samples were first purified using a microbore reversed-phase HPLC system (Magic 2002; Michrom Bioresources, CA, USA) with a Dionex (CA, USA) C18 PepMap column (150 × 1 mm i.d., 3 μm particle size, 100 Å pores) at a flow rate of 20 μL/min. Solvents A and B consisted of 95% H_2_O/5% CH_3_CN/0.1% FA/0.01% HFBA (v/v/v/v) and 95% CH_3_CN/5% H_2_O/0.1% FA/0.01% HFBA (v/v/v/v), respectively. A three-step linear gradient was used (5%-20% B in 10 min; 20%-50% B in 30 min; 50%-80% B in 20 min) for the HPLC separation. Detection was performed via a dual ultravolet/visible detector set at 220 and 280 nm. The fractions were collected manually with a fraction collector (FC 203B, Gilson, WI, USA). All the fractions were concentrated using a SpeedVac before further analysis.

### CapLC-ESI-IT MS analysis

A 5 μL aliquot of each HPLC fraction of interest was further separated using a capillary HPLC system (capLC, Waters Corporation, MA, USA) with a Dionex C18 PepMap column (150 × 0.3 mm i.d., 3 μm particle size, 100 Å pore size) at a flow rate of 2.5 μL/min. Different gradients were performed for each LC fraction using solvents A and B (A: 95% H_2_O/5% MeOH/0.1% acetic acid/0.01% TFA (v/v/v/v); B: 95% MeOH/5% H_2_O/0.1% acetic acid/0.01% TFA (v/v/v/v)). The eluent was interfaced on-line with an electrospray ionization (ESI) ion trap (IT) mass spectrometer (HCTultra PTM Discovery System, Bruker Daltonics, MA, USA). A data-dependent acquisition method was employed. The most intense ions in the MS scan were selected as precursor ions for MS/MS analysis. For each MS scan, two precursor ions were selected for fragmentation (MS/MS) based on their intensity and charge (preferably +2). The dynamic exclusion of previously fragmented precursor ions was set to 2 spectra within 1 min. Collision-induced dissociation (CID) was performed for MS/MS analysis on each precursor ion. The collision energy for CID was ramped for the most efficient and reproducible MS/MS fragmentation. The MS and MS/MS scans were performed in the range of 300-1500 and 50-2000 *m/z*, respectively.

### MALDI-TOF MS analysis

HPLC fractions were screened by a MALDI-TOF/TOF mass spectrometer (Ultraflex II; Bruker Daltonics). A 0.5 μL aliquot of each fraction, along with an equal volume of the matrix (50 mg/mL DHB in 50% CH_3_CN/50% H_2_O/0.01% TFA (v/v/v)), was spotted onto an "MTP 384 massive target T" plate (Bruker Daltonics) and air-dried. Positive-ion mass spectra were acquired using the reflectron mode within a range of 580-6000 *m/z*. The instrument was calibrated externally using a commercially available standard peptide mixture.

### Data analysis with bioinformatics tools

MS/MS data obtained from the ESI-IT MS were processed and converted to a Mascot generic file format (.mgf) using DataAnalysis software (Bruker Daltonics). The .mgf files were automatically *de novo *sequenced and then searched against the in-house zebra finch prohormone database using Peaks Studio software (Bioinformatics Solutions Inc, ON, Canada). Mass tolerance was set at ≤ 0.3 Da for MS and ≤ 0.5 Da for MS/MS. Common modifications (for example, *C*-terminal amidation, *N*-terminal pyroglutamate formation and disulfide bond) were selected as variables. The in-house database is composed of the zebra finch prohormone genes, identified by bioinformatics characterization. All obtained peptide identities were subjected to manual verification for accurate ion series, reasonable cleavage sites and PTM identification. A minimum of three consecutive ion (b- and y-ion) matches is required to be a true-positive match. Unassigned MS/MS spectra were subjected to *de novo *sequencing and subsequent BLAST search.

### *In situ *hybridization for basal expression distribution in adults

*In situ *hybridization was performed using adult males and females. Brains were flash frozen and stored at -80°C until processing. Digoxigenin-labeled riboprobes were *in vitro *transcribed from clones in the ESTIMA Songbird EST collection that mapped to prohormone genes in the zebra finch genome (http://titan.biotec.uiuc.edu/cgi-bin/ESTWebsite/estima_annotations?seqSet=songbird3; Table 3). ISH was performed as described previously [63].

In order to describe the basal distribution of prohormone gene expression, we used unmanipulated males and females removed directly from single-sex holding aviaries (*n *= 3 per sex). These brains were sectioned in the coronal plane at 18 μm and sections spanning the rostral-caudal extent of the brain were processed with ISH. We used a total of 15 ESTs for this mapping (Table 3). Eleven of the prohormone genes were selected because peptides had been identified and confirmed by MS/MS sequencing (see Additional File 1), three ESTs for peptides we were unable to confirm with MS/MS (phosphatidylethanolamine binding protein 1, orexin and cerebellin, and one was to verify the presence of the non-prohormone-derived peptide from CNP2 (see Results and Discussion section for details).

### *In situ *hybridization for song-regulated expression in adult males

We investigated prohormone genes with a functional connection to song biology and behaviour by first using sequence homology searches of the predicted prohormone gene set from the whole genome (described above) to identify the prohormone ESTs contained on the 20K Songbird Neurogenomics zebra finch brain DNA microarray [22]. We then cross-referenced these prohormone ESTs with the gene lists that showed significant changes in transcript levels in the adult male auditory forebrain after various song playback experiences [44]. Using a False Discovery Rate [45] threshold of 0.05, we identified six prohormone ESTs from this study that showed a significant change in hybridization intensities in birds with different song experiences (ADM, CCK, IGF1, GH, NTS, NPY). These ESTs were further investigated in the auditory forebrain with ISH.

We used adult males that experienced one of three acute song experiences. All birds were individually placed into acoustic chambers and exposed to novel conspecific song, familiar conspecific song, or silence (*n *= three per group) prior to sacrifice in a paradigm previously described [44]. Brains were sectioned to 12 μm in the saggital plane for focused investigation of the auditory forebrain lobule. A total of three sections representing the medial to lateral extent of the lobule were processed and analysed for each bird.

### *In situ *hybridization image capture and statistical analysis

Images were captured with either a Nikon LS-8000 slide scanner or an AxioImager A1 (Carl Zeiss Microimaging, NJ, USA) with a CCD camera (Microfire; Optronics, CA, USA). In the case of the auditory forebrain images, hybridization intensity and the number of hybridized cells above intensity threshold were quantified using ImageProPlus 4.5.1 (MediaCybernetics; MD, USA). We measured hybridization in both the auditory forebrain lobule and in the adjacent hippocampus, which does not respond to song [62]. All auditory forebrain values were normalized to the hippocampus values for statistical analysis. Normalized values for each section were summed across the three auditory forebrain lobule sections that represented one bird. These 'whole auditory forebrain lobule' ISH measurements were used for one-way ANOVA (SPSS; IL, USA) to test for differences across the song exposure conditions.

### MS analysis of peptide profiles in individual song control nuclei

Adult male brains (*n *= 2) were rapidly dissected and placed immediately into ice cold artificial cerebrospinal fluid (aCSF) for 2-5 min. Brains were then mounted and immersed in oxygenated aCSF for sectioning on a Vibratome (Vibratome 3000 Series, Ted Pella, CA, USA). Brains were cut into 500 μm slices. We visually identified slices that contained major song nuclei (Area X, LMAN, HVC and RA) and incubated them in a slice chamber (AutoMate Science, Inc., CA, USA) equipped with a temperature controller for 10 min at 41°C. The slices were continually perfused with EBSS (without phenol red), supplemented with 24.6 mM glucose, 26.2 mM NaHCO_3 _and 2.5 mg/L gentamycin, and saturated with 95% O_2_/5% CO_2 _at 45°C, pH 7.4. Song nuclei were cut out of the *ex vivo *brain slices on ice and immediately homogenized in acidified acetone (40:6:1 acetone:H_2_O:HCl, v/v/v) for MALDI-TOF MS analysis.

## Abbreviations

aCSF: artificial cerebrospinal fluid; CBLN: cerebellin; CCK: cholecystokinin; CID: collision-induced dissociation; CPN: carboxypeptidase N; ESI: electrospray ionization; EST: expressed sequence tag; FCA: fraction collector; GH: growth hormone; HPLC: high-performance liquid chromatography; ISH: *in situ *hybridization; IT: ion trap; LMAN: lateral magnocellular nucleus of the anterior nidopallium; MALDI-TOF: matrix-assisted laser desorption/ionization time-of-flight; MS: mass spectrometry; MS/MS: tandem MS; NTS: neurotensin; PEBP: phosphatidylethanolamine binding protein; PTM: posttranslational modification; RA: robust nucleus of the arcopallium; RNP: renal natriuretic peptide; SCG: secretogrann; TFA: trifluoracetic acid; VP: vasoactive intestinal peptide.

## Authors' contributions

FX, SEL and BRS contributed equally to this work. FX performed the MS experiments and analysis and wrote the manuscript; SEL performed tissue dissections, gene expression experiments and analysis and wrote the manuscript; BRS performed bioinformatic analysis and wrote the manuscript; SPA performed mass spectrometry experiments and analysis; AA contributed to the bioinformatic analysis; SLR-Z contributed to bioinformatics analysis; DFC contributed to data analysis and wrote the manuscript; JVS contributed to experimental design, data analysis and wrote the manuscript.

## Supplementary Material

Additional file 1 **The sequences and masses of identified peptides in the zebra finch by mass spectrometry**. Peptides were identified by searching the mass spectrometry (MS) data against the database of predicted prohormones in the zebra finch. Except those three marked with an asterisk (*) that were identified by mass match, all of the others were confirmed by MS/MS sequencing. Each individually detected and sequenced peptide is listed. Common posttranslational modifications were also characterized for some peptides. p- (*N*-terminal pyroglutamate formation); -amide (*C*-terminal amidation); Ac- (acetylation); ***C ***(disulfide bond). ^†^: annotated as neuropeptide based on the chicken prohormone gene database. Click here for file

Additional file 2 **Zebra finch prohormone and signaling sequences**. Predicted sequences of zebra finch prohormone and signaling genes in FASTA format. Click here for file

## References

[B1] Clayton DF Balakrishnan CN London SE Integrating genomes, brain and behavior in the study of songbirds Curr Biol 2009 19 R865 873 10.1016/j.cub.2009.07.006 19788884 PMC2890260

[B2] Adkins-Regan E Hormones and sexual differentiation of avian social behavior Dev Neurosci 2009 31 342 350 10.1159/000216545 19546571

[B3] Goodson JL Neural and peptidergic responses to conspecific stimuli vary in relation to sociality Horm Behav 2005 48 84 84

[B4] Vates GE Broome BM Mello CV Nottebohm F Auditory pathways of caudal telencephalon and their relation to the song system of adult male zebra finches (*Taenopygia guttata*) J Comp Neurol 1996 366 613 642 10.1002/(SICI)1096-9861(19960318)366:4<613::AID-CNE5>3.0.CO;2-7 8833113

[B5] Bauer EE Coleman MJ Roberts TF Roy A Prather JF Mooney R A synaptic basis for auditory-vocal integration in the songbird J Neurosci 2008 28 1509 1522 10.1523/JNEUROSCI.3838-07.2008 18256272 PMC6671566

[B6] Strand FL Neuropeptides: Regulators of Physiological Processes 1999 MIT Press

[B7] Hook V Funkelstein L Lu D Bark S Wegrzyn J Hwang SR Proteases for processing proneuropeptides into peptide neurotransmitters and hormones Annu Rev Pharmacol Toxicol 2008 48 393 423 10.1146/annurev.pharmtox.48.113006.094812 18184105 PMC2731677

[B8] Christensen D Vleck CM Prolactin release and response to vasoactive intestinal peptide in an opportunistic breeder, the zebra finch (*Taeniopygia guttata*) Gen Comp Endocrinol 2008 157 91 98 10.1016/j.ygcen.2008.04.013 18555065

[B9] Goodson JL Adkins-Regan E Effect of intraseptal vasotocin and vasoactive intestinal polypeptide infusions on courtship song and aggression in the male zebra finch (*Taeniopygia guttata*) J Neuroendocrinol 1999 11 19 25 10.1046/j.1365-2826.1999.00284.x 9918225

[B10] Fiore M Clayton NS Pistillo L Angelucci F Alleva E Aloe L Song behavior, NGF level and NPY distribution in the brain of adult male zebra finches Behav Brain Res 1999 101 85 92 10.1016/S0166-4328(98)00143-0 10342402

[B11] Kotegawa T Takahashi T Tsutsui K Ikeda T Minakata H Nomoto K Isolation and characterization of opioid peptides in the avian brain J Exp Zool 1995 273 87 95 10.1002/jez.1402730202 7595281

[B12] Bottjer SW Roselinsky H Tran NB Sex differences in neuropeptide staining of song-control nuclei in zebra finch brains Brain Behav Evol 1997 50 284 303 10.1159/000113342 9360005

[B13] Adkins-Regan E Neuroendocrinology of social behavior ILAR J 2009 50 5 14 19106448 10.1093/ilar.50.1.5

[B14] Leung CH Goode CT Young LJ Maney DL Neural distribution of nonapeptide binding sites in two species of songbird J Comp Neurol 2009 513 197 208 10.1002/cne.21947 19132730

[B15] Kabelik D Klatt JD Kingsbury MA Goodson JL Endogenous vasotocin exerts context-dependent behavioral effects in a semi-naturalistic colony environment Horm Behav 2009 56 101 107 10.1016/j.yhbeh.2009.03.017 19341739 PMC2723850

[B16] Amare A Hummon AB Southey BR Zimmerman TA Rodriguez-Zas SL Sweedler JV Bridging neuropeptidomics and genomics with bioinformatics: prediction of mammalian neuropeptide prohormone processing J Proteome Res 2006 5 1162 1167 10.1021/pr0504541 16674105 PMC2548284

[B17] Southey BR Amare A Zimmerman TA Rodriguez-Zas SL Sweedler JV NeuroPred: a tool to predict cleavage sites in neuropeptide precursors and provide the masses of the resulting peptides Nucleic Acids Res 2006 34 W267 272 10.1093/nar/gkl161 16845008 PMC1538825

[B18] Southey BR Rodriguez-Zas SL Sweedler JV Prediction of neuropeptide prohormone cleavages with application to RFamides Peptides 2006 27 1087 1098 10.1016/j.peptides.2005.07.026 16494967

[B19] Southey BR Sweedler JV Rodriguez-Zas SL A python analytical pipeline to identify prohormone precursors and predict prohormone cleavage sites Front Neuroinformatics 2008 2 7 19169350 10.3389/neuro.11.007.2008 PMC2610252

[B20] Li L Sweedler JV Peptides in our brain: mass spectrometric-based measurement approaches and challenges Annu Rev Anal Chem 2008 1 451 483 10.1146/annurev.anchem.1.031207.113053 20636086

[B21] Warren WC Clayton DF Ellegren H Arnold AP Hillier LW Kunstner A Searle S White S Vilella AJ Heger A The genome of a songbird Nature 2010 in press 10.1038/nature08819 PMC3187626 20360741

[B22] Replogle K Arnold AP Ball GF Band M Bensch S Brenowitz EA Dong S Drnevich J Ferris M George JM The Songbird Neurogenomics (SoNG) Initiative: community-based tools and strategies for study of brain gene function and evolution BMC Genomics 2008 9 131 10.1186/1471-2164-9-131 18366674 PMC2329646

[B23] Fricker LD Lim J Pan H Che FY Peptidomics: identification and quantification of endogenous peptides in neuroendocrine tissues Mass Spectrom Rev 2006 25 327 344 10.1002/mas.20079 16404746

[B24] Hummon AB Amare A Sweedler JV Discovering new invertebrate neuropeptides using mass spectrometry Mass Spectrom Rev 2006 25 77 98 10.1002/mas.20055 15937922

[B25] Perry M Li Q Kennedy RT Review of recent advances in analytical techniques for the determination of neurotransmitters Anal Chim Acta 2009 653 1 22 10.1016/j.aca.2009.08.038 19800472 PMC2759352

[B26] Svensson M Skold K Nilsson A Falth M Svenningsson P Andren PE Neuropeptidomics: expanding proteomics downwards Biochem Soc Trans 2007 35 588 593 10.1042/BST0350588 17511658

[B27] Birney E Clamp M Durbin R GeneWise and Genomewise Genome Res 2004 14 988 995 10.1101/gr.1865504 15123596 PMC479130

[B28] Delfino KR Southey BR Sweedler JV Rodriguez-Zas SL Genome-wide census and expression profiling of chicken neuropeptide and prohormone convertase genes Neuropeptides 2010 44 31 44 10.1016/j.npep.2009.11.002 20006904 PMC2814002

[B29] Kudo H Liu J Jansen EJ Ozawa A Panula P Martens GJ Lindberg I Identification of proSAAS homologs in lower vertebrates: conservation of hydrophobic helices and convertase-inhibiting sequences Endocrinology 2009 150 1393 1399 10.1210/en.2008-1301 18948394 PMC2654743

[B30] Martens G Braks J Eib D Zhou Y Lindberg I The neuroendocrine polypeptide 7B2 is an endogenous inhibitor of prohormone convertase PC2 Proc Natl Acad Sci USA 1994 91 5784 5787 10.1073/pnas.91.13.5784 8016065 PMC44081

[B31] Zhu X Lindberg I 7B2 facilitates the maturation of proPC2 in neuroendocrine cells and is required for the expression of enzymatic activity J Cell Biol 1995 129 1641 1650 10.1083/jcb.129.6.1641 7790360 PMC2291188

[B32] Lee S Lindberg I 7B2 prevents unfolding and aggregation of prohormone convertase 2 Endocrinology 2008 149 4116 4127 10.1210/en.2008-0064 18467442 PMC2488232

[B33] Yuri T Kimball RT Braun EL Braun MJ Duplication of accelerated evolution and growth hormone gene in passerine birds Mol Biol Evol 2008 25 352 361 10.1093/molbev/msm260 18048401

[B34] Bora A Annangudi SP Millet LJ Rubakhin SS Forbes AJ Kelleher NL Gillette MU Sweedler JV Neuropeptidomics of the supraoptic rat nucleus J Proteome Res 2008 7 4992 5003 10.1021/pr800394e 18816085 PMC2646869

[B35] Hummon AB Richmond TA Verleyen P Baggerman G Huybrechts J Ewing MA Vierstraete E Rodriguez-Zas SL Schoofs L Robinson GE Sweedler JV From the genome to the proteome: uncovering peptides in the Apis brain Science 2006 314 647 649 10.1126/science.1124128 17068263

[B36] Slemmon JR Blacher R Danho W Hempstead JL Morgan JI Isolation and sequencing of two cerebellum-specific peptides Proc Natl Acad Sci USA 1984 81 6866 6870 10.1073/pnas.81.21.6866 16593526 PMC392033

[B37] Morano C Zhang X Fricker LD Multiple isotopic labels for quantitative mass spectrometry Anal Chem 2008 80 9298 9309 10.1021/ac801654h 19551992 PMC2771887

[B38] Romanova EV Roth MJ Rubakhin SS Jakubowski JA Kelley WP Kirk MD Kelleher NL Sweedler JV Identification and characterization of homologues of vertebrate beta-thymosin in the marine mollusk *Aplysia californica* J Mass Spectrom 2006 41 1030 1040 10.1002/jms.1060 16924592

[B39] Hatcher NG Atkins N Jr Annangudi SP Forbes AJ Kelleher NL Gillette MU Sweedler JV Mass spectrometry-based discovery of circadian peptides Proc Natl Acad Sci USA 2008 105 12527 12532 10.1073/pnas.0804340105 18719122 PMC2518830

[B40] Hatcher NG Sweedler JV *Aplysia *bag cells function as a distributed neurosecretory network J Neurophysiol 2008 99 333 343 10.1152/jn.00968.2007 18003877

[B41] Lein ES Hawrylycz MJ Ao N Ayres M Bensinger A Bernard A Boe AF Boguski MS Brockway KS Byrnes EJ Genome-wide atlas of gene expression in the adult mouse brain Nature 2007 445 168 176 10.1038/nature05453 17151600

[B42] Derand R Montoni A Bulteau-Pignoux L Janet T Moreau B Muller JM Becq F Activation of VPAC1 receptors by VIP and PACAP-27 in human bronchial epithelial cells induces CFTR-dependent chloride secretion Br J Pharmacol 2004 141 698 708 10.1038/sj.bjp.0705597 14744818 PMC1574226

[B43] Boni LJ Ploug KB Olesen J Jansen-Olesen I Gupta S The *in vivo *effect of VIP, PACAP-38 and PACAP-27 and mRNA expression of their receptors in rat middle meningeal artery Cephalalgia 2009 29 837 847 10.1111/j.1468-2982.2008.01807.x 19220306

[B44] Dong S Replogle KL Hasadsri L Imai BS Yau PM Rodriguez-Zas S Southey BR Sweedler JV Clayton DF Discrete molecular states in the brain accompany changing responses to a vocal signal Proc Natl Acad Sci USA 2009 106 11364 11369 10.1073/pnas.0812998106 19541599 PMC2708759

[B45] Benjamini Y Hochberg Y Controlling the false discovery rate - a practical and powerful approach to multiple testing J R Stat Soc Series B Methodol 1995 57 289 300

[B46] Bottjer SW Alexander G Localization of met-enkephalin and vasoactive intestinal polypeptide in the brains of male zebra finches Brain Behav Evol 1995 45 153 177 10.1159/000113547 7796094

[B47] Blahser S Peptidergic pathways in the avian brain J Exp Zool 1984 232 397 403 10.1002/jez.1402320304 6151578

[B48] Lovell PV Clayton DF Replogle KL Mello CV Birdsong 'transcriptomics': neurochemical specializations of the oscine song system PLoS One 2008 3 e3440 10.1371/journal.pone.0003440 18941504 PMC2563692

[B49] Wada K Howard JT McConnell P Whitney O Lints T Rivas MV Horita H Patterson MA White SA Scharff C Haesler S Zhao S Sakaguchi H Hagiwara M Shiraki T Hirozane-Kishikawa T Skene P Hayashizaki Y Carninci P Jarvis ED A molecular neuroethological approach for identifying and characterizing a cascade of behaviorally regulated genes Proc Natl Acad Sci USA 2006 103 15212 15217 10.1073/pnas.0607098103 17018643 PMC1622802

[B50] Southey BR Rodriguez-Zas SL Sweedler JV Characterization of the prohormone complement in cattle using genomic libraries and cleavage prediction approaches BMC Genomics 2009 10 228 10.1186/1471-2164-10-228 19445702 PMC2698874

[B51] Richardson RD Boswell T Raffety BD Seeley RJ Wingfield JC Woods SC NPY increases food intake in white-crowned sparrows: effect in short and long photoperiods Am J Physiol 1995 268 R1418 1422 7611518 10.1152/ajpregu.1995.268.6.R1418

[B52] Gould KL Newman SW Tricomi EM DeVoogd TJ The distribution of substance P and neuropeptide Y in four songbird species: a comparison of food-storing and non-storing birds Brain Res 2001 918 80 95 10.1016/S0006-8993(01)02961-4 11684045

[B53] Wacker DW Schlinger BA Wingfield JC Combined effects of DHEA and fadrozole on aggression and neural VIP immunoreactivity in the non-breeding male song sparrow Horm Behav 2008 53 287 294 10.1016/j.yhbeh.2007.10.008 18036596

[B54] Goodson JL Evans AK Wang Y Neuropeptide binding reflects convergent and divergent evolution in species-typical group sizes Horm Behav 2006 50 223 236 10.1016/j.yhbeh.2006.03.005 16643915 PMC2570780

[B55] Goodson JL Nonapeptides and the evolutionary patterning of sociality Prog Brain Res 2008 170 3 15 full_text 18655867 10.1016/S0079-6123(08)00401-9 PMC2570786

[B56] Goodson JL Schrock SE Klatt JD Kabelik D Kingsbury MA Mesotocin and nonapeptide receptors promote estrildid flocking behavior Science 2009 325 862 866 10.1126/science.1174929 19679811 PMC2862247

[B57] London SE Dong S Replogle K Clayton DF Developmental shifts in gene expression in the auditory forebrain during the sensitive period for song learning Dev Neurobiol 2009 69 437 450 10.1002/dneu.20719 19360720 PMC2765821

[B58] Tomaszycki ML Peabody C Replogle K Clayton DF Tempelman RJ Wade J Sexual differentiation of the zebra finch song system: potential roles for sex chromosome genes BMC Neurosci 2009 10 24 10.1186/1471-2202-10-24 19309515 PMC2664819

[B59] Bolhuis JJ Gahr M Neural mechanisms of birdsong memory Nat Rev Neurosci 2006 7 347 357 10.1038/nrn1904 16760915

[B60] London SE Clayton DF Functional identification of sensory mechanisms required for developmental song learning Nat Neurosci 2008 11 579 586 10.1038/nn.2103 18391944 PMC2562764

[B61] Mello C Nottebohm F Clayton D Repeated exposure to one song leads to a rapid and persistent decline in an immediate early gene's response to that song in zebra finch telencephalon J Neurosci 1995 15 6919 6925 7472448 10.1523/JNEUROSCI.15-10-06919.1995 PMC6578026

[B62] Mello CV Vicario DS Clayton DF Song presentation induces gene expression in the songbird forebrain Proc Natl Acad Sci USA 1992 89 6818 6822 10.1073/pnas.89.15.6818 1495970 PMC49595

[B63] Jin H Clayton DF Localized changes in immediate-early gene regulation during sensory and motor learning in zebra finches Neuron 1997 19 1049 1059 10.1016/S0896-6273(00)80396-7 9390518

