# Supplementary material for: The zebra finch neuropeptidome: prediction, detection and expression
Source: BMC Biol. 2010 Apr 1;8:28. doi: 10.1186/1741-7007-8-28 (PMC2873334; doi:10.1186/1741-7007-8-28)
Supplement: Additional file 1 — The sequences and masses of identified peptides in the zebra finch by mass spectrometry. Peptides were identified by searching the mass spectrometry (MS) data against the database of predicted prohormones in the zebra finch. Except those three marked with an asterisk (*) that were identified by mass match, all of the others were confirmed by MS/MS sequencing. Each individually detected and sequenced peptide is listed. Common posttranslational modifications were also characterized for some peptides. p- (N-terminal pyroglutamate formation); -amide (C-terminal amidation); Ac- (acetylation); C (disulfide bond). †: annotated as neuropeptide based on the chicken prohormone gene database. [file 1741-7007-8-28-S1.PDF]

Additional File 1. The sequences and masses of identified peptides in the zebra finch by MS.

Prohormone	Peptide	Mass$_{obs}$	Mass$_{theo}$	Mass error (ppm)
ADCYAP1	MYTLYYPPEK	1303.57	1303.62	-38
	QMAVKKYLAAVL-amide*	1332.76	1332.78	-15
	VGGASGGLGDDAEPLT	1414.70	1414.67	21
	HIDGIFTDSYSRY	1572.70	1572.72	-13
AVP	CAEEDFLPSPCQAGGQ	1649.65	1649.68	-18
	*C*YIQN***C***PRG-amide	1049.51	1049.45	57
CCK	LDGHPVPAELE	1175.53	1175.58	-43
CRF	p-EQKSLDTDDSD	1233.81	1233.50	251
CHGA	WNKMDEL	934.45	934.42	32
	WNKMDELA	1005.53	1005.46	70
	YAFSSPEEDV	1142.53	1142.48	44
	WNKMDELAKQL	1374.71	1374.70	7
	WNKMDELAKQLT	1475.77	1475.74	20
	WNKMDELAKQLTS	1562.77	1562.78	-6
	REENDSEEDPDRSMKKTFRS	2455.10	2455.11	-4
CHGB	SAELPGLY	848.42	848.43	-12
	YDVERQY	971.53	971.43	103
	SEYRGHLPA	1028.59	1028.63	-39
	KKSHSEGMSM	1120.61	1120.50	98
	MDQLAQLLNY	1207.59	1207.59	0
	SAELPGLYSSGEE	1337.67	1337.60	52
	SEYRGHLPAEKE	1414.65	1414.68	-21
	SAELPGLYSSGEEL	1450.67	1450.68	-7
	GKTNRYDVERQY	1527.65	1527.74	-59
	SSDQVEDEEEERF	1597.61	1597.64	-19
	SSDQVEDEEEERFA	1668.11	1668.68	-342
	NPGKTNRYDVERQY	1738.73	1738.84	-63
	SSDQVEDEEEERFAE	1797.65	1797.72	-39
	DPAETEKHPAGSVEKEQ	1850.65	1850.86	-113
	SAGEQLPEGREEGRPALSE	2012.10	2011.97	65
	DSAGEQLPEGREEGRPALSE	2125.77	2125.99	-103
GH	p-QNYGLLSCFK	1154.57	1154.54	26
GNRH1	p-QHWSYGLQPG-amide	1153.57	1153.53	35
GRP	APLQPGGTPALTKIYP	1622.87	1622.90	-18
NPVF	SIKPFSNLPLRF-amide	1416.88	1416.81	49
	SPLVKGFSQSLLNLPQRF-amide	2029.00	2029.13	-64
NPY	SSPDTLISDLLL	1272.71	1272.68	24
NTS	p-QLHVN	592.21	592.30	-152
	p-QLHVNKARRPYIL[†]	1589.73	1589.92	-120
	KNPYI	633.32	633.35	-47
	KNPYIL	746.41	746.43	-27
OXT	ICCGE	523.10	523.18	-153

PENK	YGGF	442.19	442.19	0
	YGGFL	555.24	555.27	-54
	YGGFM	573.20	573.23	-52
	MDELY	669.21	669.27	-90
	YGGFMRF	876.33	876.40	-80
	YGGFMRSI	929.47	929.44	32
	YGGFMKKDSD	1146.50	1146.50	0
	ELEDEAKELQ	1202.63	1202.57	50
	SPELEDEAKEL	1258.75	1258.59	127
	RPEWWLDYQ	1291.68	1291.61	54
	ELDENHLLAKK*	1308.73	1308.71	15
	SPELEDEAKELQ	1386.63	1386.65	-14
	VGRPEWWLDYQ	1447.69	1447.69	0
	MDELYRAEPEDEANGGE	1923.61	1923.78	-88
	MDELYRAEPEDEANGGEM	2054.61	2054.82	-102
	MDELYRAEPEDEANGGEML	2167.73	2167.90	-78
	MDELYRAEPEDEANGGEMLA	2238.71	2238.94	-103
POMC	YGGFMSSE	876.30	876.40	-114
	YGGFMSSERI	1145.41	1145.52	-96
	NAIGKSFAKDGQ	1234.61	1234.63	-16
	PEFPWNSRKE	1288.57	1288.62	-39
	FKNAIGKSFAKDGQ	1509.71	1509.79	-53
	SYSMEHFRWGKPV-amide	1621.79	1621.78	6
	SYSMEHFRWGKPVG	1679.69	1679.79	-60
	EEFSGKNAGIPDFSRFSEEAKDG	2516.10	2516.15	-20
PRL	GRVHSGDVE	954.49	954.45	42
PTHLH	QNLIE	615.29	615.32	-49
RLN3	AGDLKKLVRQV	1224.80	1224.77	24
SCG2	AMENM	594.16	594.21	-84
	ASFQQHQL	957.45	957.47	-21
	PEVANTNQM	1002.39	1002.44	-50
	ASFQQHQLL	1070.67	1070.55	112
	SGKLSFLEDE	1123.57	1123.54	27
	ASFQQHQLLQ	1198.59	1198.61	-17
	EDMLAKVLEY	1208.60	1208.61	-8
	SGKLSFLEDEI	1236.65	1236.62	24
SCG5	LQHLGPYGNI	1110.67	1110.58	81
	SVPQFSDEDKGP	1304.69	1304.59	77
	SVPQFSDEDKGPK	1432.59	1432.68	-63
	SVNPYLQGQRLDNVVA	1771.87	1771.93	-34
SST	SANSNPALAPRE	1225.63	1225.61	16
TAC1	HKTDSFVGLM-amide	1132.61	1132.57	35
	RPRPQQFFGLM-amide	1374.77	1374.73	29
	SLNSGSSEGSTAQNYE	1629.55	1629.68	-80
	DAGYGQIS	809.28	809.36	-99
UCN3	VRSGDDGSGAGSHPDEASLSLLEGPE	2538.10	2538.15	-20
VIP	YLHSLIRKRVSSQDSPV*	1984.11	1984.09	10
	HSDAVFTDNYSRF	1557.57	1557.68	-71
