# Supplementary material for: The zebra finch neuropeptidome: prediction, detection and expression
Source: BMC Biol. 2010 Apr 1;8:28. doi: 10.1186/1741-7007-8-28 (PMC2873334; doi:10.1186/1741-7007-8-28)
Supplement: Additional file 2 — Zebra finch prohormone and signaling sequences. Predicted sequences of zebra finch prohormone and signaling genes in FASTA format. [file 1741-7007-8-28-S2.PDF]

Additional File 2: **Zebra finch prohormone and signaling sequences.** Predicted sequences of zebra finch prohormone and signaling genes in Fasta format. Sequence title information includes gene symbol, gene name, NCBI Entrez gene ID, Ensembl ID and Genbank predicted sequence number when available.

```
>ADCYAP1 | Adenylate cyclase activating polypeptide 1 (pituitary) | 100225028 | ENSTGUP00000010475 |
XP_002193462.1
MCSKAILALLVYGIIMHCSVYCSPAAGLQYPALRLEDEVYDEDGNTLQDFAYDQEPLGIANPSSMIGEMYTLYYPPEKRHADGIFNKAYRKLLGQLSARKYLHSLMAKRVGGA
SGGLGDDAEPLTKRHIDGIFTDSYSRYRKQMAVKKYLAAVLGKRYKQRVKNKGRRVAYL
>ADCYAP1_2 | Adenylate cyclase activating polypeptide 1 (pituitary), isoform 2  100225028 | ENSTGUP00000010475 |
XP_002193462.1
MCSKAILALLVYGIIMHCSVYCSPAAGLQYPALRLEDEVYDEDGNTLQDFAYDQEPLGIANPSSMIGEMYTLYYPPEKSGASGGLGDDAEPLTKRHIDGIFTDSYSRYRKQMA
VKKYLAAVLGKRYKQRVKNKGRRVAYL
>ADM | Adrenomedullin | 100231562 | ENSTGUP00000008261 | XP_002197355.1
MKLLHVALLYLGSVTFFGVDAARVDVATEFKRKWTKWALSRAKRDVKPAGLLRGLGAAADVMPLIRTQDVKEDSRLSPSSNREDAHIRVKRYRQSINRFPHFQTKACRFGTCT
VHWLVDELHRAAVNDRNDAAPPNKISPQGYGRRRRSLPERRSPAHSPSSGRRPRTRRALPLAAVLGV
>APLN | Apelin | na | na
>AVP | Arginine vasopressin | 100217635 | ENSTGUP00000011316 | XP_002190083.1
MAEPSLPLSFLCLLALSSACYIQNCPRGGKRALADAALRQCMPCGPGNRGNCFGPGICCGSELGCYLGTAETRRCAEEDFLPSPCQAGGQPCGAGGRCAAPGICCSAETCAMD
SACLDQGSDGAQEAAEEKNLTVLDGSAGDLLLKLMHLANRQQQQGKHPLL
>FIGF | C-fos induced growth factor (vascular endothelial growth factor D) | 100227068 | ENSTGUP00000008337 |
XP_002197500.1
MYKPWATVNIFIISFLHLLQGSDYENGSVKRTSLSALERSEQQIRRASSLEELLHITHSEDWKLWKCRLKLKSLANLDSRSASHRSTRFAAAFYDIDTLKVIDEEWQKTQCVP
RETCVEVAKELGTTTNKFFKPPCVNVFRCGGCCNEESLSCMNTSTTYVSKTLFEISVPLTSVPEPVPIKIANHTACKCTSNTQRHQYTIIRRSVQYPEEDGCPFTNKFCHNGW
IWDSDKCECVIDTQHSNRREGTPSSC
>CRF | C-RF amide peptide | 100226474 | na | XP_002199006.1
MWHLISWTPRAQWSPNHLKLVTVYVLMLLVSLSFASGQSRSFKHQIDNRSPEIDPFWYVGRGVRPIGRFGKRQLRSSHGSLRPVSRHLDFILNALWEQKSLDTDDSDW
>CNP1 | C-type natriuretic peptide 1 | 100226912 | na | XP_002197345.1
MLGLQSWPCSFFLFLVLLSASVQTVSLPGQRLQMLLSRLLPLEPKSTLTEEDTKEGSSFGPQLLSSALPFLPSGARAARPSLWRKNLSSRKWGLPGDWAWKAMPRGCFGLKLD
RIGTFSGLGC
>CNP3 | C-type natriuretic peptide 3 | na | ENSTGUP00000016882
KLLFCPGFLLLLIVSQNQAGARSISSFQSLPKLLDEDLEHPLDSEEWDHEEDDLIPAGVFDQQDTELPWIQTSREQPRSREQSRSISLGDSAIQRFFSDLLSSARRQKQKGRS
KKGLSRGCFGVRLDRIGSLSGLGC
>CALCA | Calcitonin-related polypeptide alpha | 100228652 | ENSTGUP00000008761 | XP_002198249.1
MVMLKISSFLAVYALVVCQMDSFQAAPVRPGLESITDRVTLSEYEARRLLNALVKEFIQMTAEEMEQASEGNSLDKPISKRCASLSTCVLGKLSQELHKLQTYPRTDVGAGTP
GKKRSVLSDLEHERYANYGEPLGNN
>CALCA_2 | Calcitonin-related polypeptide alpha, isoform 2 | 100228652       ENSTGUP00000008761 | XP_002198249.1
MVMLKISSFLAVYALVVCQMDSFQAAPVRPGLESITDRVTLSEYEARRLLNALVKEFIQMTAEEMEQASEGNSVTAQKRACNTATCVTHRLADFLSRSGAVGKNNFVPTNVGS
KAFGRRRRSVQI
>CARTPT | CART prepropeptide | 100229332 | na | XP_002194988.1
MDPLFGPRAGTAGASGRRLGRSGGGGAEPSRAVPCRAHTRGAAAVGADARRGGRGAIKAAAAAVERQHHGELTGAGAVRRGRRACCSLLADTERSRRASPPGRTLNTGPRGGP
SPPGGVSPSREKELVRRELGRAGAPPGRARGDGGSVVSLQIEAVQEVLEKLKSKRRPQYEKKLGQVPMCDAGEQCAVRKGARIGKLCDCPRGTSCNSFLLKCL
>CCK | Cholecystokinin | 100190220 | ENSTGUP00000004812 | XP_002199022.1
MYSGLCICVFLAVLSVSSLGQQTLGTLDGHPVPAELEQSLPEHQRHSRAPSSAGPLKPLQRLDASSDQRANIGALLAKYLQQARKGSTGRFSVMGNRVQSIDPTHRINDRDYM
```

GWMDFGRRSAEEYEYSS
>CHGA | Chromogranin A (parathyroid secretory protein 1) | 100225869 | ENSTGUP00000012975 | XP_002200181.1
MSRPELLAVLLLAVPAVSLPVTNDMNKGDTKVMKCIVEVISDTLSKPNPLPISEECLETLRGDERIISILRHQNLLKELQEIAAQGANERTQQQKKNSGFEDELSEVLESQNN
KNKQRDAAGEHPEEDQPTGSLAELAAQKPQQNEDSREEGKSLEEREPRPWDTNPVEKEDREEAESNDVRDTEDTHRSEVLHNHIGKNFSEDEQQQQGDEEEEPRGSRNSLELE
DEGEEPSRQGQEHSKEVAGERVEREDDGDEAAEEDPTEAERSLDLAEEDEEAEEMQGGDNNDDELGFGKDVRSSEEDEEEEEEEEPRALRGGRHRQEDEEMQEEEDTFHPKDA
KSDEMEEESSREWEDTKRWNKMDELAKQLTSKKRREENDSEEDPDRSMKKTFRSRKYAFSSPEEDVRRSWKHHSKEDSGEGGLPLAPMPEEKKDEEGSANRRTEDQELESLAA
IEAELERVAHKLHELRRG
>CHGB | Chromogranin B (secretogranin 1) | 100230980 | ENSTGUP00000002261 | XP_002197054.1
MGPRALLALLAAAALAGERGAGTSRRERRGANSPGSEQPREQRGRDARARSGRGRWLREPQPGVGGDGAGRELRGCSAFIFIPAQHPRTKTWRCRFLTAVLLGFCQPVLDAGR
WWQSASTVPVEKDHTEEMVTRCIVEVLSNALSKPNAPPINPECKEILKSDKNDRERSEKEQPEVRHPKDPAETEKHPAGSVEKEQRQAEEESKKYMEGGDEEKLAREEGKSEE
EEAGHHTPAQDETLHTEEKKHYQETGREEERNYHSEEESKEGRCCEDVEAAVLTKKSHSEGMSMDEFRGGNDQSPRGHWHLEEGMQSHSKQIREGKAAGKLGHHGSGAAEHSR
EDQQPQERLKLSCVLCFIKTKAPGSVQTLMREFQNGGQALLALLGVLWGAASSESREEQNKRHHQSQESSEERRQHDGAHPSEGRLYPADENQEQLARYLGEEKQRRVGGRAR
PRSREHQRSQQAAEHKGQASRHHSTEDSLEEEQELVEKKHRSSDQVEDEEEERFAERSEYRGHLPAEKEKRTAAAYRPFYPLLWWKSRHLDKRDSAGEQLPEGREEGRPALSE
KSLFPEYNDYEWWSEKQIQSALKHRRSDKRNPGKTNRYDVERQYKKMDQLAQLLNYRKKSAELPGLYSSGEELKKRPVAGNDRRSLSQRPLTEEEEKQLENLATMDLELQNIA
EKINSLRRG
>C12orf39 | Chromosome 12 open reading frame 39 (Spexin) | na | ENSTGUP00000012483
MYRCERLFLHLVVLCCAQGLHKLPASALALFLAASFIAFSWSAPQAHFQRRNWTPQAMLYLKGAQGRRFIADESQRKDIYDRVQLETRSHSTNPLSLSEAAALFLTSLQKAQE
VEEENSEYPGYL
>C2orf40 | Chromosome 2 open reading frame 40 | 100225694 | ENSTGUP00000010030 | XP_002194086.1
MPPPRPRGALPGASLLLLLFLLLPLLCAAPDVSRVNKLKLMLQKREASAAAVKPEVSVKETAAKEFLSSLRRQRRQLWDRSQPDVQQWYQQFLYLGFDEQKFEDDLSYWTNLG
RARNEYYGGYYQHHYDEDSPIGPRNPHTFRHGAGVNYDDY
>C2orf40_2 | Chromosome 2 open reading frame 40, isoform 2 | 100225694   ENSTGUP00000010030 | XP_002194086.1
MPPPRPRGALPGASLLLLLFLLLPLLCAAPASAAAVKPEVSVKETAAKEFLSSLRRQRRQLWDRSQPDVQQWYQQFLYLGFDEQKFEDDLSYWTNLGRARNEYYGGYYQHHYD
EDSPIGPRNPHTFRHGAGVNYDDY
>CRHR1 | Corticotropin releasing hormone | 100190638 | ENSTGUP00000011624 | XP_002198840.1
MKIPLLVSTGILLVALLPCQECRALSKSPAPAPGALHQPDFFQQQQRQQQQQQQQQQTLPVLLRMGEEYFLRLGNLHKRPVGSFSASSSSTSHLQPEASASNFFRAAVQQLQQ
LPERSPGDGEDGEAEGEAVEREKRSEEPPISLDLTFHLLREVLEMARAEQLAQQAHSNRKLMEIIGK
>EDN1 | Endothelin 1 | 100219314 | ENSTGUP00000005994 | XP_002187680.1
MDYCHMLVSLLFVLCPGLLPAAPGAEADAAPPPATAHRRARRCSCSSLMDEECVYFCHLDIIWINTPEKTVPYGLGGPSRPRRSLKDMVPEMLTEPSSRCRCANQKDKKCLNF
CQAGKDLWAQSTVEKTSWHRIKAGNCFGPKCMNQQLVDSKKMKRLEAIGNSIKASFSIAKLKAELQKGRKLKHNRANKRQSFRESLKAS
>EDN2 | Endothelin 2 | na | ENSTGUP00000000838
AAGLGHPPPESHLAARPRIKRCSCNSWLDKECIYFCHLDIIWVNTPGNTAPYGLGSPPRRRKRSAGRCECSHSRDSICATFCQGRPGYLQILKLPVSSGASARSLQSSATRPP
HHGLLRALRDLRVSSPRSGQRQQRSRRDTQPPALPWEKNIWKKKR
>EDN3 | Endothelin 3 | 100218153 | ENSTGUP00000008244 | XP_002195303.1
MELGLLFLFGLTITSTAGSALPRPRSALPAAGPGHRERDESGAAAAAGGRARADGGALRHRARRCTCYTYKDKECVYYCHLDIIWINTPERTVPYGLSNYRGSFRGKRSTGQT
QSAPQPSLRCSCADAHDKQCLQFCRRMQDRRRNHGSMKKAEEKDQRREEEHNFVQ
>GAL | Galanin prepropeptide | 100229595 | ENSTGUP00000010282 | XP_002199594.1
MQRCTAFLLLPLILCATLSETLGLVFSAKDKRGWTLNSAGYLLGPHAVDNHRSFNDKHGFTGKREVPPDEDIKAGNLGRPLADENIVRTVIEFLTYLHLKEVGALDKLPTPEE
INQS
>GIP | Gastric inhibitory polypeptide | na | ENSTGUP00000014511
RRYSEAALASDYSRTVEHVLGRNFVEWLLARRERDSxxxxxxxxxxxxxxxxxxxxxxxxxxxxxxxxxxxxxxxxxxxxxxxALENSAGLREFLEREFLTWLMSGELCRA
>GRP | Gastrin-releasing peptide | na | ENSTGUP00000017765
MRGGRPAALPLLALALLTAQGGAAPLQPGGTPALTKIYPRGSHWAVGHLMGKKSTGDFPYGFEEENKTPFSALPDNIKQLEEYLQWEEISKYLLTLLERNENKSGHFSKGGLP

WYTRNTWETDDNSSWKHMMDYLLQVVNMKESTPS
>GHRL | Ghrelin/obestatin prepropeptide | na | na
PTARLHEKGFKFFWGTLATEAK
>GCG | Glucagon | 100226588 | ENSTGUP00000014141 | XP_002197220.1
MKMKSIYFVAGLLLMIVQGSWQNPLQDTEEKSRSFKASHSEPIEEPRQLNEVKRHSQGTFTSDYTKYLDTRRAQDFVQWLMSTKRNGQQAQEDKENNKVPDQLSSNAISKRHA
EFERHAEGTYTSDITSYLEGQAAKEFIAWLVNGRGRRDFPEKALLAEEMGRRHADGTFTSDINKVLDDMAAKEFLKWLINTKVTQRDLLEEYQ
>GCG_2 | Glucagon, isoform 2 | 100226588 | ENSTGUP00000014141 | XP_002197220.1
MKMKSIYFVAGLLLMIVQGSWQNPLQDTEEKSRSFKASHSEPIEEPRQLNEVKRHSQGTFTSDYTKYLDTRRAQDFVQWLMSTKRNGQQAQEDKENNKVPDQLSSNAISKRHA
EFERHAEGTYTSDITSYLEGQAAKEFIAWLVNGRGRRE
>CGA | Glycoprotein hormones, alpha polypeptide | 100227314 | ENSTGUP00000012836 | XP_002198312.1
MDCYGKYAAVTLTILSVFLHLLHTFPDGEFLMQGCPECKLGENRFFSKPGAPIYQCTGCCFSRAYPTPMRSKKTMLVPKNITSEATCCVAKAFTKITLKDNVKIENHTDCHCS
TCYYHKS
>GNRH1 | Gonadotropin-releasing hormone 1 (luteinizing-releasing hormone) | 100216318 | ENSTGUP00000004488 |
XP_002197436.1
MEKPRRMVATALLCVLAVGLGLAQHWSYGLQPGGKRSTQLLLGPFQEIANEMEKLEEEQQSACPGSHQNSRSGALREAMERLLEEQGRRKNV
>GH1 | Growth hormone 1 | 100218808 | ENSTGUP00000003400
MARGSWLSPLAIAVITLGLQWPQAAATFPAMPLSSLFANAVLRAQHLHLLAAETYKEFERSYIPEDQRHTNKNSQVAYCYSETIPAPTGKEDAQQKSDMELLQFSLVLIQSWL
TPVQYLSKVFTNNLVFGTSDRVYEKLKDLEEGIQALMRELEERSPRGPQVLKPTYEKFELHLRGEEALVKNYGLLSCFKKDLHXVETYLKVMRCRRYGEGNCAL
>GH1A | Growth hormone 1 (duplicate on Chr1) | 100232594 | ENSTGUP00000013961
MAPGSWFPPLTIAMITLGLQWPQQAAAFPAIPLSSLFTNAVLRAQHLHLLAAETYKEFERTYIPEDQRHTNKNSQVAFCYSEAIPAPVKKDDAQQKSDIELLQFSLVLIQSWL
TPVQYLSKMFTNNLVFGTSDRVYEKLKDLEEGIQALMRELQDRSFRGPQILKATYEKFDIHLRSEDALLQNYGLLSCFKKDLHKVETYLKVMKCRRYGEGNCTI
>GHRH | Growth hormone releasing hormone | 100218168 | ENSTGUP00000005071 | XP_002190265.1
MQRHADAIFTDNYRKFLAQISARKFLQTIIGKRLGSSKSSPGEGVQEFLSRRQSDSILMDSQYQQQMVLRDFLGAILQNQRPQDIDSSGLEGFPSTLAKLM
>HCRT | Hypocretin (orexin) neuropeptide precursor | 100225610 | ENSTGUP00000018297 | XP_002197774.1
MEVPNSKPRRAACLLLLLLLCSLAAARHSLPHCCRQKTCPCRVYDLLHGMGNHAAGILTLGKRKSVPLTFQSRLYRLLHGSGNHAAGILTMGKRGQQPGTVCHDSPGCPAGTG
AQPTAAPRGAATSPDGPRECQGHSGKDLSTGQAQGAARSFY
>INS | Insulin | 100231606 | ENSTGUP00000009610 | XP_002198969.1
MALWIRSLPLLALLAVSGPGSSHGAVNQHLCGSHLVEALYLVCGERGFFYQPKARRDVEQPLVSGPLHGELGELPFQQEEFETVKRGIVEQCCHNTCSLYQLENYCN
>INSL5 | Insulin-like 5 | na | ENSTGUP00000010477
MRGTVLALGCLALLLLLARGGEGEGNKVRLCGRDFIRAVVFTCGGSRWRRHQDLRGSENPQRFLHEDEDDADFSSHPEPRLGTHREPLDVKPEQELQNSSKVSVLHKREAAEL
LATSCCNVGCSRTEISSLC
>IGF1 | Insulin-like growth factor 1 (somatomedin C) | 100226283 | ENSTGUP00000011633 | XP_002199849.1
MEKINSLSTQLVKCCLCDFLKVKMHTVSYIHFFYLGLCLLTLTSSVAAGPETLCGAELVDALQFVCGDRGFYFSKPTGYGSSSRRLHHKGIVDECCFQSCDLRRLEMYCAPIK
PPKSARSVRAQRHTDMPKAQKEVHLKNTSRGNTGNRNYRM
>IGF2 | Insulin-like growth factor 2 (somatomedin A) | 100144432 | ENSTGUP00000009618 | NP_001116438
MCAARRMLLLLLAFLAYAMDSAAAYGTAETLCGGELVDTLQFVCGDRGFYFSRPVGRNNRRFNRGIVEECCFRSCDLALLETYCAKSVKSERDLSATSLAGLPALSKESFQKP
SHGKYSKYDVWQKKSSQRRQRDAPNILRARRYRWQVEGLQAAEEAKALHRPLISLPSQRPPAARASPETAGPQK
>IAPP | Islet amyloid polypeptide | 100230121 | ENSTGUP00000012072 | XP_002197698.1
MCNVRLSVFFIALSVTLSCLEATPIESHQLEKRKCNTATCVTQRLADFLVRSSSSLGALYPPTNVGSNTYGKRDTAGLPSTQPQNRARL
>IAPP_2 | Islet amyloid polypeptide, isoform 2 | 100230121 | ENSTGUP00000012072 | XP_002197698.1
MCNVRLSVFFIALSVTLSCLEATPIERLLSVADDLSDGTSKRQGWMLPVMSQNSLSALREEMPEHPAAKTKSHQLEKRKCNTATCVTQRLADFLVRSSSSLGALYPPTNVGSN
TYGKRDTAGLPSTQPQNRARL
>MLN | Motilin | 100217914 | ENSTGUP00000001847

MVSRKVVASLLLVSLLSVLAEQTQGFMPFFTQSDFQKMQEKERNKAGQKKSLSSLQQLEEEGFSEQSGVDANAAKTLQQALPVRAWLTPRQLEKYQDVLEKLLAELLQDTPD
>NPPA | Natriuretic peptide precursor A | 100217781 | ENSTGUP00000016155 | XP_002191085.1
MDTKGSFFYGFLLLLLIQLQSSRANPIYSLSPAKELASMEALLERLEDKFAMMEALESNPDLQEPKTQEEILSELADDSDNQKAEPRLAPNTPLSYRDPFLKRLRGLQMPRMM
RDSGCFGRRIDRIGSLSGMGCNGSRRN
>NMB | Neuromedin B | 100222573 | ENSTGUP00000004989
MALRCLLLLLCGAALGPAVHLDFAEHRSQAAKIKVNPRGNLWATGHFMGKKSVTGSPHLETAEEPAVPMVFGPSLRALLEDMMELLTRELLKILLQERLLDENQGKYDLTGQE
TGLLTKVLEKYFSS
>NMU | Neuromedin U | na | ENSTGUP00000007709
MGHLWHPQPPAAAPERSPRHGADGAALPGSPLLLLCLLLASSIPACRGAPMPSQALQSDEDLQLLKEIDDACSAYLSTDSQPQVSSTLEELCFLIMGFFQKPQDLDEKDNTKR
FLFHYSKTHDSGNSDVMSSVLHPLLQLVPHLNERRLKRYKLDEELQGPGGIQSRGYFFYRPRNGRRSVDFR
>NPS | Neuropeptide S | na | ENSTGUP00000011907
WGQVPTAKVQITSKGKPEYFLVLLSSCLSKVGRGEELVLGKPLPESLFHKRSFRNGVGAGIKKTSFRRAK
>NPVF | Neuropeptide VF precursor (Gonadotropin-inhibitory hormone) | 100038821 | ENSTGUP00000002951 |
XP_002193656.1
MKVISNKKLILFALATVVFLTSNSMCLNEPMKSRLQSREDNDDKYYEIKDNILEEKQRSLNLEEMEDWRSKDIIKMNPFAASKMPNSVANLPLRFGRNYPEERSIKPFSNLPL
RFGRAFGENIRNHAPKVSHRLERSPLVKGFSQSLLNLPQRFGKSLPVNLPRDVEEFEPGI
>NPW | Neuropeptide W | na | na
GEAVAGVPGAGHSPARSLPCAMARGQLSGGAWGALVLLGLMLPAAPAGAWYKHVASPRYHTVGRASGLLMGVRRSPYLWRRELPAEPLRYPGTSGGDSPPSPGQSPPAAGPAP
PPPGPGRLLQRLLRRGWGW
>NPY | Neuropeptide Y | 100232180 | ENSTGUP00000002889 | XP_002193048.1
MTHRMQMQGTVRPWESVLTFALSLMVCLGTLAEAYPSKPDSPGEDAPAEDMARYYSALRHYINLITRQRYGKRSSPDTLISDLLLRESTENIPRSRFEDPSMW
>NTS | Neurotensin | 100190409 | ENSTGUP00000008164 | XP_002196110.1
MRVQLVCVVLLALASCSLCSDSEEEMKALEADLLTNMYTSKINRAKLPYWKMTLLNVCNLVNNINNQMGETVEVDEEDLVPGRQFPAALDGFSLEAMLTVYQLQKVCHSRAFQ
HWELLQQDALDLENSSQEKEIMKRKNPYILKRQLHVNKSRRPYILKRSSYY
>OSTN | Osteocrin | 100230749 | ENSTGUP00000009566 | XP_002190638.1
MLQFQLVVVHVALVIALLWWDSSSVLLAQAAPEPLEPSAALGVGDHPIASEEKPATNLAAKLFLLDDLVSLENEVTETKKKRSFPGFGSPIDRISSSSVDAKGKQRKVFELPK
RRFGVPLDRIGVSHLDNTRG
>OXT | Oxytocin, prepropeptide | 100220528 | ENSTGUP00000011315 | XP_002190044.1
MSCKALALCLLGLLALSSACYIQNCPIGGKRAVLDMDIRKCLPCGPRDKGRCFGPNICCGEELGCHIGTSDTLRCQEENFLPTPCESGHKACGSGGSCAAPGICCSTEGCGTD
SSCDQEMLFV
>PPY | Pancreatic polypeptide | na | na
MAPPLPLLLLLCAAALLPERPRAAPVHPAFPGPEAPAEELLRFYNDLQQYLNVVTRPR
>PTH | Parathyroid hormone | 100221585 | ENSTGUP00000016235 | XP_002187569.1
MTSIRNLAQAAIILCAMCFFTTSDGRPMVRRSVSEMQLMHNLGEHRHTVERQDWLQMKLQDVHSALEDARTQRPRSKDDVVLGRRLLPEHPRAAGQKKSMDLDKAYMDVLFKT
KP
>PTHLH | Parathyroid hormone-like hormone | 100230763 | ENSTGUP00000013060 | XP_002198366.1
MFAKLFQQWSFAVFLLSYSVPSYGRSVEGISRRLKRAVSEHQLLHDKGKSIQDLRRRIFLQNLIEGVNTAEIRATSEVSPNPKPATNTKNYPVRFGSEDEGRYLTQETNKSQT
YKEQPLKVSGKKKKAKPGKRKEQEKKKRRTRSAWLNSGMYGDIVTESPLLDISVTTHNQTLRRR
>PDGFA | Platelet-derived growth factor alpha polypeptide | 100222155 | ENSTGUP00000008778 | XP_002191527.1
MSGCSRHLVPIRAGGTGAISRRGGEEGGCEGKASCPRGRTGAGCGARDGLRGARAGCGVRLRLRRIGYDDVSETNLRSYSVHSAKHVQENRPVPIRRKRSIEEAIPAVCKTRT
VIYEIPRSQIDPTSANFLIWPPCVEVKRCTGCCNTSSVKCQPSRIHHRSVKLHF
>PDGFB | Platelet-derived growth factor beta polypeptide (simian sarcoma viral (v-sis) oncogene homolog) |
100228287 | ENSTGUP00000010592 | XP_002195507.1

```
MTVQECIGGYEPAQRWCRHPSWSCLECALRVYESAPGDHSYPVFADHLSNACFSLSCLFVVWQGDPIPEDIYEILGGSSVRSISDLQRALQIDSVERDSSSLSLNASQPDQNP
VALSRERRSLDALAAAETAVLAECKTREVVFEISRNMVDSTNANFVVWPPCVEVQRCSGCCNNRNVQCRPTQIRVRHVQVNKIEFVQRKPKFTKVVVPLEDHVQCRCEVVFRP
PPRNIRPGPREQRRLSPALTTAAVSQRRRVRRPPAQKRKHKKYKHVNDKKVLKEILIA
>PDGFD | platelet derived growth factor D | 100221512 | ENSTGUP00000013067 | XP_002199932.1
MQLLILLLLYAVVCANFCSRQGTTAPAQSGSIKALRASNIRRDESNHLTDLYRKEETISVAGNGCIHSPRFPSSYPRNLLLTWRLHSPESTRIQLAFDNQFGLEEPENEICRY
DFVEVEDVSETSTVIRGRWCGHKEVPPRITSRTNHIKITFKSDDYFVAKPGFKICYSLVDDFQHAASETNWESVTSSVSGVSYPSPSVTDPTLTAEALDQTIAAFDTVEDLLK
HFNPDSWQEDLENLYTDSGHHYRGRSYHDRKSKVDLDRLNDDVKRYSCTPRNYSVNLREELKLTNVVFFPRCLLVQRCGGNCGCGTPNWKSCTCVSGKTVKKYHEVLKFIPEA
GHARRRGRIRNNMSLVDIQLDHHERCDCVCSSRPPR
>PNOC | Prepronociceptin | na | na
MRSVLWELLLLLWLCARARGDCRGECLSCKRRLEGGGFDGLICLLECEGRAVPRATWELCAASSRPAPRPRRIPSPV
>PMCH | Pro-melanin-concentrating hormone | 100223423 | ENSTGUP00000011636 | XP_002199866.1
MCISSYILILSLLSLFSQGFLLSRSLQEAEDEDMLLAALNLGKILQNGDKSTSRGAIPLLKHYKTEDSSVFNDKNAGNMKVLDRGSRHDFFSHVKPINLGRKQLPYPALKGAM
AFPADTEFQNIESIEERETTDEENSSKFPIGRRDFDILRCMLGRVYRPCWQV
>PDYN | Prodynorphin | na | na
RDGDEEPTGAGAEGPELAQLHKRYGGFLRRIRPKLKWDNQKRYGGFLRRQFKVTTRSDEDPSAYSGEVSDL
>PENK | Proenkephalin | 100190068 | ENSTGUP00000011453 | XP_002198192.1
MALLLRLGCSLLALSACLLPRARADCGRDCAACAYRLGPRAGIHPLACTLECEGKLPSAKAWETCKELLQLTKLDLSEDGNIAPGDKKELDENHLLAKKYGGFMKRYGGFMKK
MDELYRAEPEDEANGGEMLAKRYGGFMKKDSDDDALANSSDLLKELLGTGDSPEAAHYREINENDGDVSKRYGGFMRSIKRSPELEDEAKELQKRYGGFMRRVGRPEWWLDYQ
KRYGGFLKRFADSILPSEEDGETYSKEVPEMEKRYGGFMRF
>PROK2 | Prokineticin 2 | na | ENSTGUP00000010242
LLLLPAHLPSDITRACERDQQCGRGMCCAVSLWIRSLRMCTPMGNLGDECHPLSHRVPFSGRRMHHTCPCLPGLACLRTPHSRFRCLPDFRKEDVFF
>PRL | Prolactin | na | ENSTGUP00000006544
MSTKGASLKGLLLAALLVSHMLLTKEGVTSLPICPNGSVNCQLSLEELFDRAVKLSHYIHFLSSEMFNEFDERYAQGRGFIAKAVNSCHTASLTTPEDKEQAQQIHHEDLLNL
ILGVLRSWNDPLIHLASEVQRIKEAPETILWKAVEIEEQNKRLLEGMEKIVGRVHSGDVENDVYTPWDGLPSLQLADEDSRLFAFYNLLHCLRRDSHKIDNYLKVLKCRLIHD
NNC
>PRLB | Prolactin B | na | ENSTGUP00000006179
AALALLWLLVCAPGDAGCHPLTVADLFDRVIRHSGRIHSLSTALYAELEKHFPSRDNELGRPARKCHTSGMLTPNGKEYAQKIPREELTQVILKLLQAWKEPLSHFNQHIEHH
QELPDDSLSKAKQISNMVHELKTGVEKVTEKMQSMGIISNSLNGMASSEGTGLSISNEANMMSDSDFIHCFRRDSNKVQSYLKILKCRIMPENSC
>PRLH | Prolactin releasing hormone | na | na
MKLGVACLLWLLLVFLTLPASHGRVRERSMEIRNPDIDPSWYTGRGIRPVGRFGRRQA
>POMC | Proopiomelanocortin | 100219426 | ENSTGUP00000017296
MCSLLLLPLLLLLGPAPGGSERCRERPECRPLPIPACPGWCRAGISSESPLFPGNGQFQPLSESLRRYVMSHFRWNQFGRKNASEPGKREEFSGKNAGIPDFSRFSEEAKDGK
RSYSMEHFRWGKPVGRKRRPVKVYPNGAEEESEENSRLEFRRGEASQEEDEDEEEEFPEFPWNSRKEKRYGGFMSSERIRTPLVTLFKNAIGKSFAKDGQ
>QRFP | Pyroglutamylated RFamide peptide | 100222487 | na | XP_002195395.1(partial)
MRAPYSLSCLFLLSLGACIPPGERWEPAEPGEGAALGGGWQRAAEGRGARRRRSEELEALLSIARELRGYSAAGAGQRPGGSGGPGALPVVGEKRSGTLGNLAEEINGYNRRK
GGFTFRFGR
>RLN3 | Relaxin 3 | 100225505 | ENSTGUP00000005181 | XP_002193109.1
MFPLSGAPLHQRVGTLGSTDSGAPDSAQTASNKLLGSFNLQSVLDPEVEQLQRSSPFLGWETFKDLYSLNYYNEYVPVAGDLKKLVRQVEEAVQKDRGGTGNENPMESSSYLW
ARYPRRKRESLGLAGMCCKWGCTKAEISTICRV
>SCT | Secretin | 100219077 | ENSTGUP00000007375 | XP_002196651.1
MVSMMTTLWQVIIPIIVLSHFSASLPSQERMKRHADGLFHSELSKMNGNAYVQQLVKHLVGLKDRSLRHSDGLFTSEYSKMRGNAQVQKFIQNLMGRKRSSPGPVNTDMQARE
GVNKPEELCFLWLYQSFLNTSHSDRDAREAAAITSQYLCPFSKQLVADMKEDMDGSE
>SCG2 | Secretogranin II (chromogranin C) | 100219534 | ENSTGUP00000008135 | XP_002198010.1
```

```
MAETKTFQPGAACALTFFFVLICWVDAASFQQHQLLQKDPDYAMKNLQRLPNPDMIKALEYIEDLRKQTNKGESSPDYSSYQSVPYLLPQRESKDQLHLPDNVWDSLTEDESQ
WVKVMLEALRQAEKESKAGPKENKPYGLSSDNNFPAGVTDDYEAYKWPERWQKYLKMPLGHYEDSSRDSPFKRTNEIVEEQYTPQSLATLESVFQELGKMAGPSNHKKERLDE
DQKLYTDDEDDVYKVNNIAYEDVVGGEDWNPIEEKVESQTQEEIKDSKEEIDKHEEEIDEEMKRSGKLSFLEDEIRRENKDQVSEDVSKLMNYYLKRLMGSAGNRKLRTGGEL
EEKRASMFLDKQLDPQAIAQLIEISRNLQIPPEDLIDMLKAGEKKQLQSERLEAEQEMEFPEDLDEITETNLGQSDIFKNNINSKNGYMKQPLIPENLPEDLNIEDIVSLLGN
DNLANQNPSYLLNRLNQGNDLPRLSYIPRRLKGHLFPKAAWMNDLERRQTEYEKLNEKDEELADYLAKVLAKYPEVANTNQMKRAPAAASESKLQEEEHLEQAIREHLNQLGP
QEAAKLASLSKRLSMAGEADDTQTRQYLDEDMLAKVLEYLKQEKSELERDHITKRAMENM
>SCG5 | Secretogranin V (7B2 protein) | 100228713 | ENSTGUP00000012091
MKVAATMITTLLCNMVFLLAFGLASAFSHSPRTPDRVTEADIQRLLHGVMEQLGIARPRVEYPAHQATNLVGPQSIEGGAHEGLQHLGPYGNIPNIVAELTGDNVPKDFSEDQ
GYPDPPNPCPIGKTVDDGCLEDTPDTAEFSREYQLHQNLFDPEHDYPNRGKWSKSLLFEKINGGPKRRKRSVNPYLQGQRLDNVVAKKSVPQFSDEDKGPK
>SST | Somatostatin | 100219295 | na
MLSCRLQCALALLSIALALGTVSAAPSDPRLRQFLQKSLAAAAGKQELAKYFLAELLSEPSQTENEALESEDLSRGAEQDEVRLELERSANSNPALAPRERKAGCKNFFWKTF
TSC
>SST2 | Somatostatin 2 | 100221267 | ENSTGUP00000017225 | XP_002186566.1
MQLVASLASVLLLLWSVRATALPGQEGLQSTREQSAARKDVILKMLAGLLGSVDVGAEVAFPAGQEKTRLEEDQAALGRLAQLSQRDRKAPCKNFFWKTFTSC
>TAC1 | Tachykinin, precursor 1 | 100227778 | ENSTGUP00000001703 | XP_002197434.1
MRLPLAFAVLLLASSQALGEEMGATDDLSYWSDWSDSDQAKEELPLPLEHFLQRMARRPRPQQFFGLMGKRDAGYGQISHKRHKTDSFVGLMGKRSLNSGSSEGSTAQNYERR
RK
>TAC1_2 | Tachykinin, precursor 1, isoform 2 | 100227778 | ENSTGUP00000001703 | XP_002197434.1
MRLPLAFAVLLLASSQALGEEMGATDDLSYWSDWSDSDQAKEELPLPLEHFLQRMARRPRPQQFFGLMGKRDAGYGQISHKRSSEGSTAQNYERRRK
>TSHB | Thyroid stimulating hormone beta | 100220136 | ENSTGUP00000000984 | XP_002197026.1
MSPFFVLSLLFGLIFGQTASLCAPSEYTIHVEKRECAYCLAINTTICAGFCMTRDSNGKKLLLKSALSQNVCTYKEMLYRTALIPGCPHHTIPYYSYPVAVSCKCGKCNTDYS
DCVRERVRTNYCTKPQKLCNL
>TRH | Thyrotropin-releasing hormone | 100228179 | ENSTGUP00000010894 | XP_002189664.1
MSSIQLPLLLLCLTSCGVCFNGGHLLPEESENMGKVPLDDTLQRSESLILQSVLKKAEKEEEINKELNAPLLQRLSKRQHPGEKYLNNLKKRQHPGKRDVEEETFYGDIQKRQ
HPGKREMEDDLDVYLELKRQQPPGIKSLLDQFAYSPRAQLTYMNELSKREHLGRRYLMFKHQHPSKRGWNYEVDVYGEKRQHPGKRHWNFDRSDDTGPCNFQESFTCHKGSLL
LDLVEDVSRDRVEEKRQHPGKRSAWESETEE
>UCN | Urocortin | na | na
MRVRMISAASVLVLLFLPSETCSPLQWPRGPSRRLTLAPQLTWEPWMGAPRPPVPATDPLPQRLCLFHGAELGPAPRARRALQTGRRRDGKPNSLDLTFHLLREFLEMSREER
LAQKALSNKLLLQSIGK
>UCN3 | Urocortin 3 | 100221165 | na | XP_002193953.1
MCSPQAPLATLRGRMLLSFLLLLGTPTRVWKGQSHKWLPPPPQAVDGGKLMRQDSTSPSKMLPDPSKVRSGDDGSGAGSHPDEASLSLLEGPERQALPWLMSPATKRAAPRKN
GRKVSLSFDVHTHLLKILLDLAREKELQAKAAANAELMARLGRRR
>UTS2 | Urotensin 2 | 100222633 | ENSTGUP00000002613 | XP_002195510.1
MNKLILCCLIIVSFSCPLLSLPIINASEMSYQHSADEDSRLNLERLGSTSLLQLLPELLGTLTEDSRAGLTPSNYNPGENIKETFHGNHPRNAFLGRFLIKDRKQYKKRGNLS
ECFWKYCV
>UTS2D | Urotensin 2 domain containing | 100218300 | ENSTGUP00000009562 | XP_002190569.1
MWSAQLCLGVLTILTMALCVPSTHGDPFLLQGKLEYRLHDTENRVLPEREDTNHENTLLTLLLNKKFAWRRPESIDWELAKKFEELEELEKLKDQLSAEDGSEVAYALESLSA
SQPKKRACFWKYCI
>UTS2D_2  Urotensin 2 domain containing, isoform 2   100218300       ENSTGUP00000009562 | XP_002190569.1
MWSAQLCLGVLTILTMALCVPSTHGDPFLLQENRVLPEREDTNHENTLLTLLLNKKFAWRRPESIDWELAKKFEELEELEKLKDQLSAEDGSEVAYALESLSASQPKKRACFW
KYCI
>VEGFC | Vascular endothelial growth factor C | 100221203 | ENSTGUP00000006624 | XP_002189592.1
MHLLEVLSLSCCLAAGAVLLGPRQPAAAAAYESGQGYYEEEPDAGEAKAHGSKELEEQLRSVSSVDELMTVLYPEYWKIFKCQLRRGGWQHNREHSSFDTRSDDSLKFAAAHY
```

NAEILKSIDTEWRKTQCTPREVCVDVGKEFGATTNTFFKPPCVSIYRCGGCCNSEGLQCMNISTNYISKTLFEITVPLSHGPKPVTVSFANHTSCRCMSKLDVYRQVHSIIRR
SLPAAQTQCHVANKTCPKNHIWNNQICRCLSQHEFGFSSHLGDSDTPEGFHICGPNKELDEETCQCVCKGGVRPSSCGPHKELDRTSCQCMCKNKLIPASCGPNKEFDEEKCQ
CVCRKTCPRHQPLNPAKCVCECIESPNKCFLKGKRFHHQTCSCYRPPCTVRTKRCDAGFYFSEEVCRCVPTYWKRPLMN
>VIP | Vasoactive intestinal peptide | 100217965 | ENSTGUP00000011533 | XP_002187804.1
MSSASSILYTVSFSNRLGNRMPFDGASEPDHARGSLKSESDILQNTLPENEKFYFDLSRIIDRNARHADGIFTSVYSHLLAKLAVKRYLHSLIRKRVSSQDSPVKRHSDAVFT
DNYSRFRKQMAVKKYLNSVLTGKRSQEELNPAKLRDEAEHLEPSFSENYDAVDELLSHLPLDL
>VIP_2 | Vasoactive intestinal peptide, isoform 2 | 100217965 | ENSTGUP00000011533 | XP_002187804.1
MEHRGASPLLLALALLSALCWRARALPPRGAAFPPVPRLGNRMPFDGASEPDHARGSLKSESDILQNTLPENEKFYFDLSRIIDSSQDSPVKRHSDAVFTDNYSRFRKQMAVK
KYLNSVLTGKRSQEELNPAKLRDEAEHLEPSFSENYDAVDELLSHLPLDL
